# Supplementary material for: Piezoelectricity and topological quantum phase transitions in two-dimensional spin-orbit coupled crystals with time-reversal symmetry
Source: Nat Commun. 2020 May 8;11:2290. doi: 10.1038/s41467-020-16058-2 (PMC7210280; doi:10.1038/s41467-020-16058-2)
Supplement: Supplementary file 1 — Supplementary Information [file 41467_2020_16058_MOESM1_ESM.pdf]

Piezoelectricity and Topological Quantum Phase Transitions in Two-Dimensional Spin-Orbit Coupled Crystals with Time-Reversal Symmetry

Yu et al.

Supplementary Materials For "Piezoelectricity and Topological Quantum Phase Transitions in Two-Dimensional Spin-Orbit Coupled Crystals with Time-Reversal Symmetry"

Jiabin Yu[1] and Chao-Xing Liu[1,*]

[1]*Department of Physics, the Pennsylvania State University, University Park, PA, 16802*

This supplementary materials include details on piezoelectric tensors (PETs), the effective models for the gap closing, and the two realistic material systems.

Contents

1. Derivation of the PET 2

2. Details on PET for Each PG 3
- A. PG $p1$ 3
- B. PGs $p1m1$, $c1m1$ and $p1g1$ 3
  - **I.** Scenario (i): TRIM 4
  - **II.** Scenario (ii): $\mathcal{U}\in\mathcal{G}_0$ but $\mathcal{T}\notin\mathcal{G}_0$ 4
  - **III.** Scenario (iii): $\mathcal{UT}\in\mathcal{G}_0$ but $\mathcal{T}\notin\mathcal{G}_0$ 5
  - **IV.** Scenario (iv): trivial $\mathcal{G}_0$ 5
- C. PG $p3$ 6
  - **I.** Scenario (i):TRIM 6
  - **II.** Scenario (ii): $C_3\in\mathcal{G}_0$ and $\mathcal{T}\notin\mathcal{G}_0$ 9
  - **III.** Scenario (iii): trivial $\mathcal{G}_0$ 9
- D. PG $p31m$ and PG $p3m1$ 9
  - **I.** Scenario (i): TRIM 10
  - **II.** Scenario (ii): $\mathcal{U}\in\mathcal{G}_0$ and $\mathcal{T}\notin\mathcal{G}_0$ 11
  - **III.** Scenario (iii): $\mathcal{UT}\in\mathcal{G}_0$ and $\mathcal{T}\notin\mathcal{G}_0$ 11
  - **IV.** Scenario (iv): trivial $\mathcal{G}_0$ 11
- E. 10 PGs with 2D Inversion or C_2 12

3. Numbers of FTPs and Effective Models for the Gap Closing 12
- A. PG $p1$ 12
  - **I.** Not TRIM 12
  - **II.** TRIM 13
- B. $p1m1$, $c1m1$, and $p1g1$ 13
  - **I.** Scenario (i): TRIM 13
  - **II.** Scenario (ii): $\mathcal{U}\in\mathcal{G}_0$ but $\mathcal{T}\notin\mathcal{G}_0$ 13
  - **III.** Scenario (iii): $\mathcal{UT}\in\mathcal{G}_0$ but $\mathcal{T}\notin\mathcal{G}_0$ 14
- C. p_3 14
  - **I.** Scenario (i):TRIM 14
  - **II.** Scenario (ii): $C_3\in\mathcal{G}_0$ and $\mathcal{T}\notin\mathcal{G}_0$ 14
- D. $p31m$ and $p3m1$ 15
  - **I.** Scenario (i): TRIM 15
  - **II.** Scenario (iii): $\mathcal{UT}\in\mathcal{G}_0$ and $\mathcal{T}\notin\mathcal{G}_0$ 15

4. VCN in Tight-Binding Model 15

5. (111) HgTe/CdTe Quantum Well 17
- A. d-induced PET jump for $\mathcal{E}=0$ 17
- B. $\mathcal{E}$-induced PET jump for fixed d 17
- C. Projection of the Kane Model 18
- D. Construction of the Hamiltonian based on symmetry 19

6. $BaMnSb_2$ 21
A. Review 21
B. TB Calculation of PET 22
C. Effective Model Analysis 22

References 23

Supplementary Note 1. Derivation of the PET

In this section, we derive Eq. (3) in the main text via linear response theory from Eq. (2) in the main text, which is equivalent to the derivation in Supplementary Ref. [1]. The derivation is done with the natural unit $c = \hbar = 1$ and the metric $(-, +, +)$.

To apply the linear response theory, we start from an action S that includes the electronic effective model and the leading order effect of the infinitesimal strain. Since the current is present in Eq. (2) , we should include the $U(1)$ gauge field that accounts for the electromagnetic field. With the $U(1)$ gauge field, the action reads

$$S = \int \frac{d^3k}{(2\pi)^3}\psi_k^\dagger G_0^{-1}(k)\psi_k + \int \frac{d^3k}{(2\pi)^3}\int \frac{d^3q}{(2\pi)^3}\left[\psi^\dagger_{k+q/2}\frac{\partial G_0^{-1}(k)}{\partial k^\mu}\psi_{k-q/2}eA^\mu(q) - \psi^\dagger_{k+q/2}M_{ij}\psi_{k-q/2}u_{ij}(q)\right] , \tag{1}$$

where $k^\mu = (\omega, \mathbf{k})_\mu$, A^μ and u_{ij} and ψ follow the same Fourier transformation rule, $G_0(k) = [\omega - h_0(\mathbf{k})(1 - \mathrm{i}\epsilon)]^{-1}$ is the time-ordered Green function without the electron-strain coupling, the chemical potential is chosen to be the zero energy, and M_{ij} is the matrix coupled to the strain tensor u_{ij}. To the leading order, the linear response is given by the following effective action

$$S_{eff} = \int d^3x\ e\partial_\nu A_\mu u_{ij} f^{ij,\mu\nu} , \tag{2}$$

where

$$f^{ij,\mu\nu} = -\frac{1}{2}\int \frac{d^3k}{(2\pi)^3}\{\mathrm{Tr}[G_0\frac{\partial G_0^{-1}}{\partial k_\mu}G_0\frac{\partial G_0^{-1}}{\partial k_\nu}G_0 M_{ij}] - (\mu \leftrightarrow \nu)\} , \tag{3}$$

and the absence of the Chern-Simons term AdA is due to the $\mathcal{T}$ symmetry.

With Supplementary Eq. (2) and Eq. (2) , we can use the condition that u_{ij} is uniform to derive the expression of the PET, resulting in

$$\gamma_{ijk} = -ef^{jk,i0} . \tag{4}$$

To further derive Eq. (3) , we define $h(\mathbf{k}, u_{ij}) = h_0(\mathbf{k}) + u_{ij}M_{ij}$ and $G(k, u_{ij}) = [\omega - h(\mathbf{k}, u_{ij})(1 - \mathrm{i}\epsilon)]^{-1}$ as the Hamiltonian and Green function with the electron-strain coupling, respectively. Using $\partial_{k_\mu}G^{-1} = \partial_{k_\mu}G_0^{-1}$ and $\partial_{u_{ij}}G^{-1} = -M_{ij}$, we can revise Supplementary Eq. (4) to

$$\gamma_{ijk} = \frac{e}{2}\int \frac{d^3k}{(2\pi)^3}\{\mathrm{Tr}[G\frac{\partial G^{-1}}{\partial k_i}G\frac{\partial G^{-1}}{\partial \omega}G\frac{\partial G^{-1}}{\partial u_{jk}}] - (k_i \leftrightarrow \omega)\}|_{u_{ij}\to 0} . \tag{5}$$

Define $X^\mu = (\omega, k_i, u_{jk})$ and then the above equation can be further transformed to

$$\gamma_{ijk} = -\frac{e}{3!}\int \frac{d^2k d\omega}{(2\pi)^3}\epsilon^{\mu\nu\rho}\,\mathrm{Tr}[G\frac{\partial G^{-1}}{\partial X^\mu}G\frac{\partial G^{-1}}{\partial X^\nu}G\frac{\partial G^{-1}}{\partial X^\rho}]\Big|_{u_{ij}\to 0} , \tag{6}$$

where $\epsilon^{\mu\nu\rho}$ is the Levi-Civita symbol. Integrating out ω in the above equation with the Wick rotation gives Eq. (3) . Although the derivation here is done for $\hbar = c = 1$, all the expressions of γ_{ijk} and the resultant Eq. (3) stay the same after converting to the SI unit as they carry the right unit for the PET in 2+1D.

Finally, we would like to discuss the effect of the identity term of h_0 in Supplementary Eq. (2) when h_0 is a two band model. In general, the Hamiltonian can always be split into the identity part and the traceless part as $h_0(\mathbf{k}) = m_0(\mathbf{k})\mathbb{1} + h_0^{\text{traceless}}(\mathbf{k})$. The eigenvalues of $h_0(\mathbf{k})$ then read $m_0(\mathbf{k}) \pm \varepsilon(\mathbf{k})$, where $\pm\varepsilon(\mathbf{k})$ are two eigenvalues of $h_0^{\text{traceless}}(\mathbf{k})$ with $\varepsilon(\mathbf{k}) > 0$ chosen without loss of generality. As the model is gapped and the Fermi energy ($E = 0$) is chosen to lie inside the gap, we have $\varepsilon(\mathbf{k}) > |m_0(\mathbf{k})| \geq 0$. Since the poles of G_0 are at $\omega = [m_0(\mathbf{k}) \pm \varepsilon(\mathbf{k})](1 - \mathrm{i}\epsilon)$, integrating ω along $(-\infty, \infty)$ in $f^{ij,\mu\nu}$ of Supplementary Eq. (2) gives the same result as integrating ω along $(-\infty + m_0(\mathbf{k})(1-\mathrm{i}\epsilon), \infty + m_0(\mathbf{k})(1-\mathrm{i}\epsilon))$ owing to the absence of poles in between the two paths. As a result, we can directly neglect the identity term of a two-band insulating h_0 in $f^{ij,\mu\nu}$ of Supplementary Eq. (2).

Supplementary Note 2. Details on PET for Each PG

The discussion on the electronic effective model and FTP of the gap closing between two non-degenerate states has some overlap with Supplementary Ref. [2]. However, the topological property and PET jump of the gap closing between two insulating states have not been discussed in Supplementary Ref. [2].

A. PG $p1$

In the main text, the effective Hamiltonian for scenario (ii) of $p1$ is derived in a non-Cartesian coordinate system, which is not convenient for the generalization to other PGs with more crystalline symmetries. Thus, we re-derive the effective Hamiltonian in the Cartesian coordinate system, as given by (see Supplementary Note **3** A)

$$\begin{aligned} h_\pm(\mathbf{q}, u) =& E_0(\pm\mathbf{q})\sigma_0 + (v_x q_x + v_0 q_y)\sigma_x + v_y q_y \sigma_y \\ & \pm m\sigma_z + \xi_{0,ij}\sigma_0 u_{ij} \pm \xi_{a',ij}\sigma_{a'} u_{ij} \ . \end{aligned} \tag{7}$$

Here we only perform the unitary transformation on the bases of the Hamiltonian and do not rotate the momentum or the coordinate system. Correspondingly, the PET jump across the direct TQPT at $m = 0$ can be derived as

$$\begin{aligned} \Delta\gamma_{xij} &= -e\frac{\Delta N_+}{\pi}\frac{\xi_{y,ij}}{v_y} \\ \Delta\gamma_{yij} &= e\frac{\Delta N_+}{\pi}\left(\frac{\xi_{x,ij}}{v_x} - \frac{v_0}{v_x}\frac{\xi_{y,ij}}{v_y}\right) \ . \end{aligned} \tag{8}$$

Supplementary Eq. (7)-(8) resemble the conclusion for $p1$ in the Results and are useful for the discussion of the other 6 PGs with non-vanishing PET.

We would like to discuss more about the GC and PET for $p1$. In the first scenario, all TRIM have no essential differences and the gap closing always happens between two Kramers pairs unless more parameters are finely tuned. Therefore, there is no need to further classify this scenario into finer cases, and the codimension for the gap closing is 5, indicating that this scenario cannot be direct TQPT[3]. According to the main text, no finer classification is needed for the second scenario either, the codimension of the gap closing scenario is 1, and it is indeed a direct TQPT that changes the Z_2 index and leads to the PET jump.

B. PGs $p1m1$, $c1m1$ and $p1g1$

In this part, we study three PGs, $p1m1, c1m1$, and $p1g1$, all of which are generated by a mirror-related symmetry $\mathcal{U}$ and the lattice translation. $\mathcal{U}$ is a mirror operation for $p1m1/c1m1$ and a glide operation for $p1g1$. The difference between $p1m1$ and $c1m1$ lies on the directions of the primitive lattice vectors relative to the mirror line, which is not important for our discussion here. Without loss of generality, we choose the mirror or glide line to be perpendicular to x, labelled as m_x or g_x, respectively. The glide operation is thus denoted as $g_x = \{m_x|0\frac{1}{2}\}$, where "$0\frac{1}{2}$" represents the translation by half the primitive lattice vector along y. The $\mathcal{U}$ symmetry in these three PGs requires $\gamma_{xxx} = \gamma_{xyy} = \gamma_{yxy} = \gamma_{yyx} = 0$, whereas the PET components $\gamma_{xxy}, \gamma_{xyx}, \gamma_{yxx}, \gamma_{yyy}$ are allowed to be nonzero. For the symmetry analysis here, the PET behaves the same under the glide and mirror operations since u_{ij} is considered in the continuum limit.

In order to classify the gap closing scenarios, we define the group $\mathcal{G}_0$ for a gap closing momentum $\mathbf{k}_0$ such that $\mathcal{G}_0$ contains all symmetry operations that leave $\mathbf{k}_0$ invariant. Since $\mathcal{G}_0$ can include the TR-related operation, it can

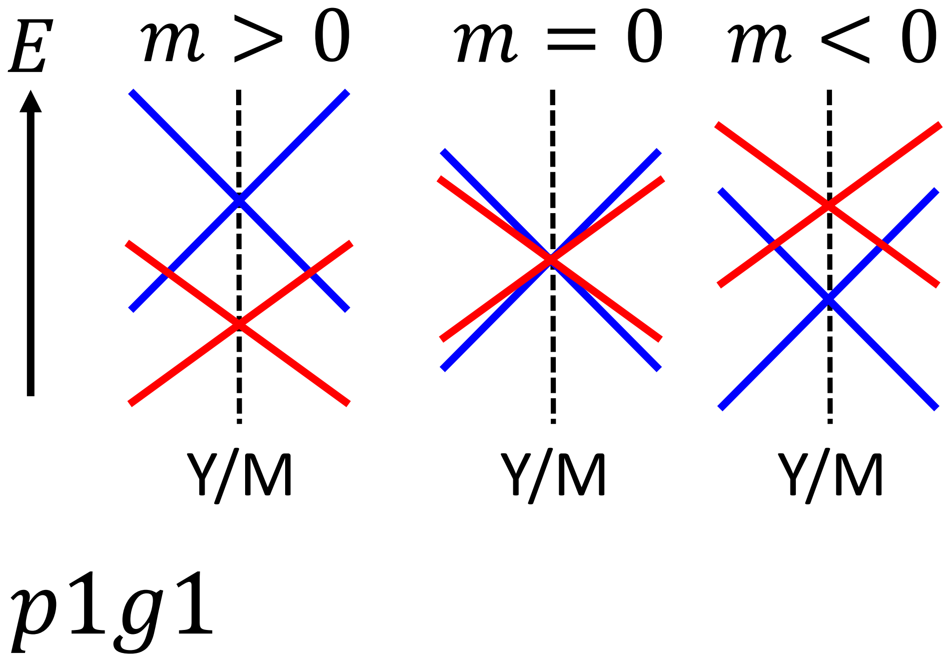

Supplementary Fig. 1. The figure shows the gap closing at Y or M in scenario (i) for $p1g1$. The lines indicate the band dispersion along k_y, and those in the same (different) colors have the same (opposite) g_x eigenvalues. m labels the gap at Y or M, and when the gap at Y or M is open ($m \neq 0$), the system is still in a gapless phase.

be larger than the little group of $\mathbf{k}_0$. Based on $\mathcal{G}_0$, we obtain in total 4 gap closing scenarios for these three PGs: (i) the gap closing at TRIM ($\mathcal{G}_0$ contains $\mathcal{T}$), (ii) $\mathcal{G}_0$ contains $\mathcal{U}$ but not $\mathcal{T}$, (iii) $\mathcal{G}_0$ contains $\mathcal{UT}$ but not $\mathcal{T}$, (iv) $\mathcal{G}_0$ contains no symmetries other than the lattice translation, which we refer to as the trivial $\mathcal{G}_0$. As summarized in Tab. 1 in the main text, the TQPT exists in scenario (iii) and (iv), which can lead to the jump of symmetry-allowed PET components.

I. Scenario (i): TRIM

In scenario (i), the gap closing requires 3 (5) fine-tuning parameters (FTPs) for $p1m1$ and $c1m1$ if m_x is (is not) in the $\mathcal{G}_0$. (See Supplementary Note **3** B.) For $p1g1$, the TRIM (Γ, X, Y and M) are split into two classes according to the value of g_x^2: Γ, X with $g_x^2 = -1$ and Y, M with $g_x^2 = 1$. The gap closing at Γ, X needs 3 FTPs since g_x behaves the same as m_x, while the gap closing at Y, M needs only 1 FTP if it happens between two Kramers pairs with opposite g_x eigenvalues. However, such gap closing at Y, M is in between two g_x-protected gapless phases with codimension 0, where the bands with opposite g_x eigenvalues cross with each other at momenta other than Y, M as shown in Supplementary Fig. **1**. Therefore, there is no direct TQPT between two gapped phases in scenario (i).

II. Scenario (ii): $\mathcal{U} \in \mathcal{G}_0$ but $\mathcal{T} \notin \mathcal{G}_0$

The same situation occurs for scenario (ii). In scenario (ii), the gap closes at two different momenta $\pm\mathbf{k}_0$ that are invariant under the $\mathcal{U}$ operation, meaning that the bases at $\pm\mathbf{k}_0$ can have definite $\mathcal{U}$ eigenvalues. The gap closing between the two bases with the same $\mathcal{U}$ eigenvalues requires 2 FTPs, as discussed in Supplementary Note **3** B. When the gap closes between two bands with opposite $\mathcal{U}$ eigenvalues, the system always enters a stable $\mathcal{U}$-protected gapless phase with 0 codimension. (This case is not the same as the scenario (i) since only one side is guaranteed to be gapless.) Thus, the gap closing cases cannot be direct TQPTs.

III. Scenario (iii): $\mathcal{UT} \in \mathcal{G}_0$ but $\mathcal{T} \notin \mathcal{G}_0$

In scenario (iii), the gap closing occurs at two different momenta $\pm\mathbf{k}_0$ that are invariant under $\mathcal{UT}$, as shown by the orange dashed lines in Fig. 1 (b) and (c) in the main text.

For $p1m1$ and $c1m1$ ($\mathcal{U} = m_x$), $(m_x\mathcal{T})^2 = 1$ suggests that we can have $m_x\mathcal{T} \doteq \mathcal{K}$ at $\mathbf{k}_0$ by choosing the appropriate bases and the band touching point at $\mathbf{k}_0$ should typically occur between two non-degenerate bands. We further take $\mathcal{T} \doteq \mathrm{i}\sigma_y\mathcal{K}$ by choosing the appropriate bases at $-\mathbf{k}_0$, and thus the two-band effective models $h_\pm(\mathbf{q}, u)$ at $\pm\mathbf{k}_0$ can be given by Supplementary Eq. (7) with extra constraints

$$v_0 = \xi_{a_1,xy} = \xi_{a_1,yx} = \xi_{y,xx} = \xi_{y,yy} = 0 \tag{9}$$

for $a_1 = 0, x, z$. As a result, only 1 FTP m is needed for the gap closing ($m = 0$), and only one single Dirac cone exists in half 1BZ at the transition, leading to the change of the Z_2 index. Based on Supplementary Eq. (8), the jump of symmetry-allowed PET components across this TQPT can be derived as

$$\begin{aligned}
\Delta\gamma_{xxy} &= \Delta\gamma_{xyx} = -e\frac{\Delta N_+}{\pi}\frac{\xi_{y,xy}}{v_y} \\
\Delta\gamma_{yxx} &= e\frac{\Delta N_+}{\pi}\frac{\xi_{x,xx}}{v_x} \\
\Delta\gamma_{yyy} &= e\frac{\Delta N_+}{\pi}\frac{\xi_{x,yy}}{v_x}\ .
\end{aligned} \tag{10}$$

For $p1g1$ with $\mathcal{U} = g_x$, since $(g_x\mathcal{T})^2 = 1$ at $(k_x, 0)$ and $(g_x\mathcal{T})^2 = -1$ at $(k_x, \pm\pi)$, we have two different gap closing cases. When the gap closes at $(\pm k_{0,x}, 0)$, the algebra relation involving $g_x\mathcal{T}$ is the same as $m_x\mathcal{T}$, *e.g.* $(g_x\mathcal{T})^2 = (m_x\mathcal{T})^2 = 1$, and thus the effective Hamiltonian can be chosen to be the same as that for $p1m1$ and $c1m1$, leading to 1 FTP, Z_2 index change, and the same form of PET jump. On the contrast, due to $(g_x\mathcal{T})^2 = -1$ at $(\pm k_{0,x}, \pm\pi)$, the gap closing needs 4 FTPs and thus no TQPT can occur in this case. (See Supplementary Note **3** B.)

IV. Scenario (iv): trivial $\mathcal{G}_0$

In scenario (iv), the gap should close simultaneously at four momenta $\mathbf{k}_0$, $\mathbf{k}_1 = -\mathbf{k}_0$, $\mathbf{k}_2 = \mathcal{U}\mathbf{k}_0$, and $\mathbf{k}_3 = -\mathcal{U}\mathbf{k}_0$, as depicted in Fig. 1 (d) and (e) in the main text. The gap closing at $\mathbf{k}_0$ can be described by the Hamiltonian $h_+(\mathbf{q}, u)$ in Supplementary Eq. (7), and the Hamiltonian at $\mathbf{k}_1$, $\mathbf{k}_2$, and $\mathbf{k}_3$ can be given by $\mathcal{T}h_+(-\mathbf{q}, u)\mathcal{T}^\dagger$, $\mathcal{U}h_+(\mathcal{U}^{-1}\mathbf{q}, \mathcal{U}^{-1}u(\mathcal{U}^{-1})^T)\mathcal{U}^\dagger$, and $\mathcal{UT}h_+(-\mathcal{U}^{-1}\mathbf{q}, \mathcal{U}^{-1}u(\mathcal{U}^{-1})^T)(\mathcal{UT})^\dagger$, respectively. Therefore, the gap closing can be achieved by tuning 1 FTP, *i.e.* m in $h_+(\mathbf{q}, u)$, in this scenario.

There is no change of Z_2 index for this scenario, since two Dirac cones exist in half 1BZ when the gap closes and the CN of contracted half 1BZ can only change by an even number. Nevertheless, scenario (iv) can still be "topological" in the context of valley Chern number (VCN) as elaborated in the following. Due to the Dirac Hamiltonian form shown in Supplementary Eq. (7), the Berry curvature is peaked at each valley $\mathbf{k}_{0,1,2,3}$ for a small m and can be captured by the electronic part of the corresponding effective Hamiltonian. Then, we can integrate the Berry curvature given by the effective model and get the VCN [4,5] for each valley as $N_{\mathbf{k}_i} = -\eta_i \mathrm{sgn}(v_x v_y)\mathrm{sgn}(m)/2$ with $i = 0, 1, 2, 3$. The values of η_i at different valleys are related by the TR and $\mathcal{U}$ symmetries, both of which flip the sign of the Berry curvature. Thus, we have $\eta_0 = \eta_3 = 1$ and $\eta_1 = \eta_2 = -1$. It should be pointed out that the Berry curvature integral is not over the entire 1BZ and the VCN at each valley thus does not need to be an integer. Nevertheless, *the change of VCN* across the gap closing is defined on a closed manifold and must be an integer number, given by $\Delta N_{\mathbf{k}_i} = -\eta_i \mathrm{sgn}(v_x v_y)$ as varying m from 0^- to 0^+. For the convenience of further discussion, we can define the VCN of the whole system[5] as $N_{val} = \sum_i \eta_i N_{\mathbf{k}_i} = -2\mathrm{sgn}(v_x v_y)\mathrm{sgn}(m)$, and the change of the VCN becomes $\Delta N_{val} = -4\mathrm{sgn}(v_x v_y) = 4\Delta N_+$ with the factor 4 for the four valleys. Therefore, if we restrict all the valleys to be far apart in the momentum space, the change of the VCN is a well-defined topological invariant and this gap closing scenario is a TQPT.

In principle, tuning parameters may merge different valleys at some high symmetry momentum, *e.g.* the valleys at $\mathbf{k}_0$ and $\mathcal{U}\mathbf{k}_0$ merged at the mirror or glide line. Therefore, without the constraint of well-defined valleys, two phases with different VCNs can share the same band topology and thus can be adiabatically connected. It means the topology characterized by VCN is "locally stable"[6], though globally unstable. Nevertheless, we restrict all valleys to be well-defined in our discussion and refer to the gap closing scenario as a TQPT.

Next we study the change of the PET components at this TQPT, which can be split into two parts: $\Delta\gamma^{(0)}$ originating from $\pm\mathbf{k}_0$ and $\Delta\gamma^{(1)}$ given by $\pm\mathcal{U}\mathbf{k}_0$. $\Delta\gamma^{(0)}$ equals to Supplementary Eq. (8) since the effective models at $\pm\mathbf{k}_0$ are the same as Supplementary Eq. (7). Owing to the mirror or glide symmetry, $\Delta\gamma^{(1)}$ is related to $\Delta\gamma^{(0)}$ as

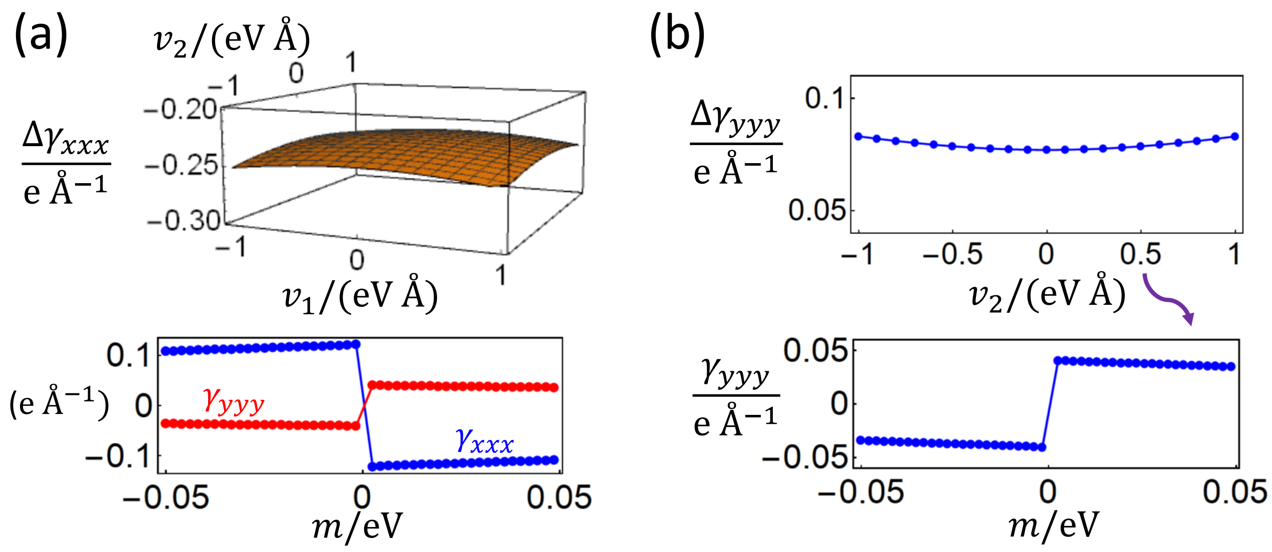

Supplementary Fig. 2. The upper panels of (**a**) and (**b**) numerically show the PET jump induced by the gap closing at Γ point as the function of $v_{1,2}$ and v_2, respectively. The lower panels of (a) and (b) plot the PET component as a function of m for $(v_1, v_2) = (0.5, 0.5)$eVÅ and $v_2 = 0.5$eVÅ, respectively.

$\Delta\gamma^{(1)}_{ijk} = (\mathcal{U})_{ii'}(\mathcal{U})_{jj'}(\mathcal{U})_{kk'}\gamma^{(0)}_{i'j'k'}$. As a result, we obtain the non-zero jump of symmetry-allowed PET components $\Delta\gamma_{ijk} = \Delta\gamma^{(0)}_{ijk} + \Delta\gamma^{(1)}_{ijk}$ as

$$\begin{aligned}
\Delta\gamma_{xxy} &= \Delta\gamma_{xyx} = -e\frac{\Delta N_{val}}{2\pi v_y}\xi_{yxy} \\
\Delta\gamma_{yxx} &= -e\frac{\Delta N_{val}(-v_y\xi_{xxx} + v_0\xi_{yxx})}{2\pi v_x v_y} \\
\Delta\gamma_{yyy} &= -e\frac{\Delta N_{val}(-v_y\xi_{xyy} + v_0\xi_{yyy})}{2\pi v_x v_y} \ .
\end{aligned} \tag{11}$$

C. PG $p3$

PG $p3$ is generated by 3-fold rotation C_3 and the lattice translation. Owing to C_3, the PET only has two independent components γ_{xxx} and γ_{yyy} as

$$\begin{aligned}
\gamma_{yxy} &= \gamma_{yyx} = \gamma_{xyy} = -\gamma_{xxx} \\
\gamma_{xxy} &= \gamma_{xyx} = \gamma_{yxx} = -\gamma_{yyy} \ .
\end{aligned} \tag{12}$$

Again, we classify the gap closing for $p3$ according to $\mathcal{G}_0$, resulting in three different scenarios: (i) $\mathcal{G}_0$ contains $\mathcal{T}$, (ii) $\mathcal{G}_0$ contains C_3 but not $\mathcal{T}$, and (iii) $\mathcal{G}_0$ is trivial. Here we do not have a scenario for $\mathcal{G}_0$ containing $C_3\mathcal{T}$ but no $\mathcal{T}$, since $(C_3\mathcal{T})^3$ is equivalent to $\mathcal{T}$. As summarized in Tab. 1 in the main text and elaborated in the following, in any of the above scenarios, there are gap closing cases between gapped states that need only 1 FTP, change the Z_2 index, and lead to the discontinuous change of symmetry-allowed PET components.

I. Scenario (i):TRIM

There are 4 TRIM in scenario (i), namely three M points related by C_3 and one Γ point, as labeled in Fig. 1 (f) in the main text. $\mathcal{G}_0$ of each individual M point only contains $\mathcal{T}$ and the lattice translation, and thus the gap closing at

M needs 5 FTPs, same as the gap closing at TRIM for $p1$.

When the gap closes at Γ point as shown in Fig. 1 (f) in the main text, $\mathcal{G}_0$ also contains C_3 with $C_3^3 = -1$. Due to $[C_3, \mathcal{T}] = 0$, the Kramers pairs can be classified into two types according to the C_3 eigenvalues: one with $(e^{-i\pi/3}, e^{i\pi/3})$ and the other with $(-1, -1)$. The gap closing between the Kramers pairs of the same type requires more than 1 FTPs, 3 for $(e^{-i\pi/3}, e^{i\pi/3})$ type and 5 for $(-1, -1)$ type, as discussed in Supplementary Note **3** C.

The gap closing with 1 FTP happens between the TR pairs of different types, for which the minimal four-band effective Hamiltonian in the bases $(e^{-i\pi/3}, e^{i\pi/3}, -1, -1)$ reads

$$h_{p3}(\mathbf{k}, u) = h_{p3,0}(\mathbf{k}) + h_{p3,1}(u) \ , \tag{13}$$

where $h_{p3,0}$ is the electron part

$$\begin{aligned} h_{p3,0}(\mathbf{k}) = & E_0\tau_0\sigma_0 + m\tau_z\sigma_0 + (v_1k_x + v_2k_y)(\frac{\tau_0+\tau_z}{2})\sigma_x \\ & + (v_1k_y - v_2k_x)(\frac{\tau_0+\tau_z}{2})\sigma_y + (v_3k_x + v_4k_y)\tau_x\sigma_z \\ & + (-v_3k_y + v_4k_x)\tau_y\sigma_0 + (v_5k_x + v_6k_y)\tau_x\sigma_x \\ & + (-v_5k_y + v_6k_x)\tau_x\sigma_y \ , \end{aligned} \tag{14}$$

and $h_{p3,1}$ describes the electron-strain coupling

$$\begin{aligned} h_{p3,1}(u) = & (u_{xx} + u_{yy})(\xi_1\tau_0\sigma_0 + \xi_2\tau_z\sigma_0) \\ & + (-u_{xx} + u_{yy})(\xi_3\tau_y\sigma_z + \xi_5\tau_y\sigma_x - \xi_4\tau_x\sigma_0 + \xi_6\tau_y\sigma_y) \\ & + (u_{xy} + u_{yx})(\xi_4\tau_y\sigma_z + \xi_6\tau_y\sigma_x + \xi_3\tau_x\sigma_0 - \xi_5\tau_y\sigma_y) \ . \end{aligned} \tag{15}$$

τ's and σ's are Pauli matrices that label two different Kramers pairs and two components of each Kramers pair, respectively, m is the gap closing tuning parameter, and the bases are chosen such that $\mathcal{T} \doteq -i\tau_0\sigma_y\mathcal{K}$.

This gap closing is certainly a TQPT since it changes the numbers of IRs of the occupied bands, meaning that the two gapped states separated by this gap closing cannot be adiabatically connected. When $v_1 = v_2 = 0$, we can define an effective inversion symmetry $\widetilde{P} = \tau_z\sigma_0$ for the electron part of Supplementary Eq. (13), $\widetilde{P}h_{p3,0}(-\mathbf{k})\widetilde{P}^\dagger = h_{p3,0}(\mathbf{k})$, and thus the gap closing of $h_{p3,0}(\mathbf{k})$ with $v_1 = v_2 = 0$ changes the Z_2 index according to the Fu-Kane criteria[7] since the parity of the occupied band changes. The existence of non-zero v_1, v_2 terms that break $\widetilde{P}$ cannot influence the Z_2 topology change, since (i) the Z_2 topology does not rely on the effective inversion symmetry, and (ii) additional gap closing away from Γ is forbidden at $m = 0$ as long as the $v_{1,2}$ terms are restored adiabatically. Therefore, within a certain range of $v_{1,2}$, the codimension-1 gap closing at $m = 0$ is a direct TQPT that changes the Z_2 index.

The remaining question is if the codimension-1 gap closing at $m = 0$ is always a Z_2 transition. To answer this question, note that we can always assume the transition at $m = 0$ is Z_2 for a parameter region S_1 of v_i's in $h_{p3,0}$ and non-Z_2 for the other parameter region S_0 of v_i's. Since the same form of the Hamiltonian can not correspond to Z_2 and non-Z_2 transitions simultaneously, the intersection of S_0 and S_1 is empty. Now we suppose both S_0 and S_1 are codimension-0 subspaces of the v_i parameter space (not the whole parameter space since only v_i's are included while m is excluded). Then, the boundary of S_0, labeled as ∂S_0, is a codimension-1 subspace of v_i parameter space, and the special transition at $(m = 0, v_i \in \partial S_0)$ is a codimension-2 transition, as shown in Supplementary Fig. **3**.

Patching the Hamiltonian with $m = \pm\epsilon$ with ϵ positive and infinitesimal gives a Hamiltonian that lives on a closed manifold. This Hamiltonian has Z_2 trivial and nontrivial ground states when v_i's are in S_0 and S_1, respectively, as shown in Supplementary Fig. **3**. As v_i's change from S_0 to S_1 passing through ∂S_0, the patched Hamiltonian must experience a gap closing at generic k points that changes Z_2 (Supplementary Fig. **3**), since there is always an energy gap at Γ for $m \neq 0$. As discussed with more detail in the following (see scenario (iii)), the gap closing at generic k points surely changes the Z_2 index, is codimension-1, and simultaneously happens at six momenta. The gap closing can only happen either for $m = \epsilon$ part or for $m = -\epsilon$ part of the patched Hamiltonian but not both, since if the gap closes twice, the Z_2 index would be changed back. It means, the codimension-1 hypersurface for the gap closing at generic k (red line in Supplementary Fig. **3**) touches the codimension-1 hypersurface for gap closing at Γ ($m = 0$ line in Supplementary Fig. **3**) just from one side of m but not passing through. As mentioned above, the touching part at $(m = 0, v_i \in \partial S_0)$ is a codimension-2 transition, owing to the assumption that both S_0 and S_1 are codimension-0 subspaces of v_i parameter space.

At the touching, we must have the six generic gap closing points merging at Γ. Otherwise, we should expect the red line in Supplementary Fig. **3** to pass through the $m = 0$ line instead of stopping, since the gap closing process is local in the momentum space and different gap closing cases cannot influence each other if they happen at the different momenta. However, the merging process cannot be codimension-2 since moving a generic gap closing point

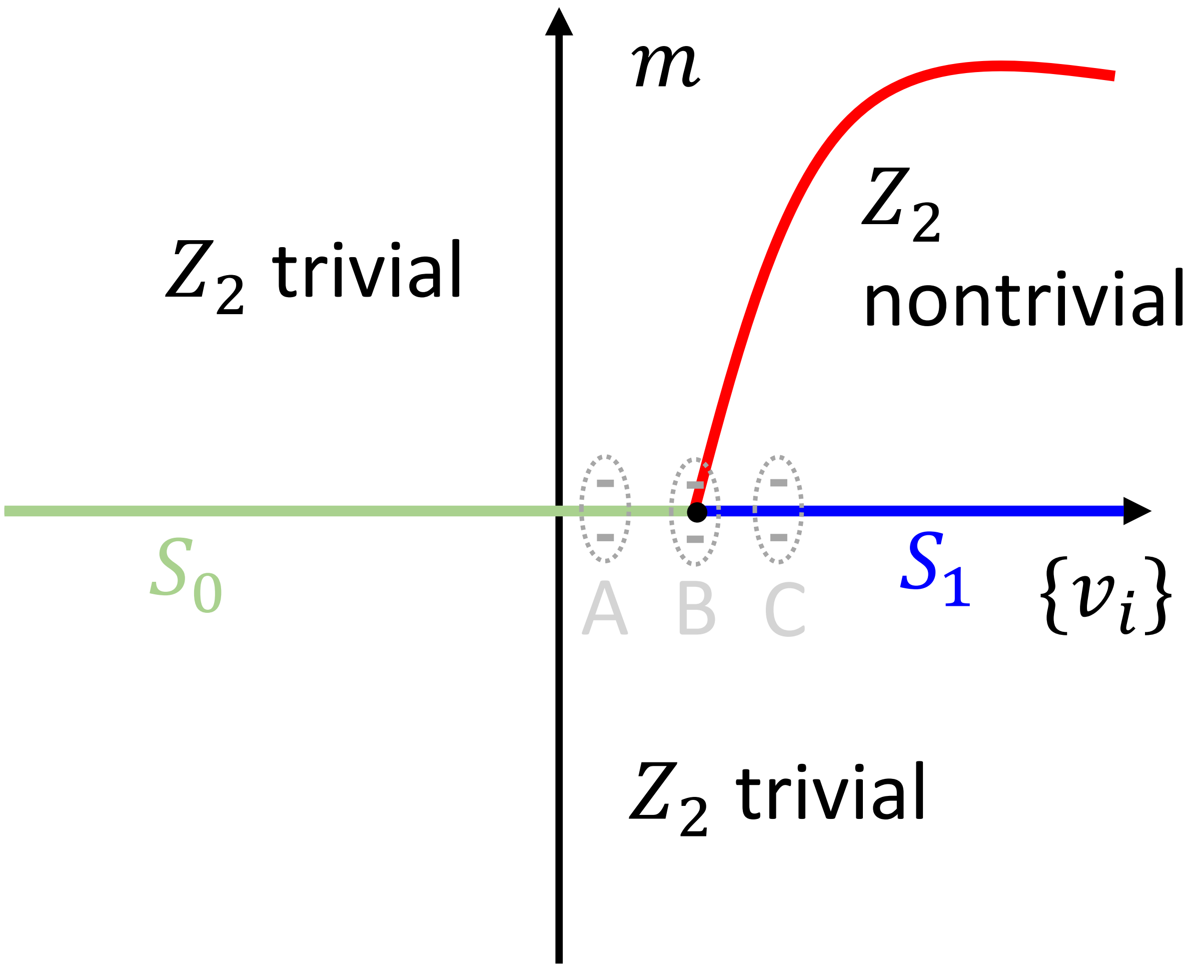

Supplementary Fig. 3. This figure shows the typical phase diagram around $(m = 0, v_i \in \partial S_0)$ (the black dot), within the assumption that both S_0 and S_1 are codimension-0 subspaces of the v_i parameter space. The green line is $(m = 0, v_i \in S_0)$ which does not change Z_2 index, the blue line is $(m = 0, v_i \in S_1)$ that changes Z_2 index, and the system closes the gap at six generic k points on the red line. Without loss of generality, we choose the red line to touch the $m = 0$ line from the positive m side. A,B, and C label three different Hamiltonians given by patching the two effective models with $m = \pm\epsilon$ at different values of v_i. A is Z_2 trivial, C is Z_2 non-trivial, and B closes the gap at the six generic k points for in $m = \epsilon$ part.

to a specific momentum while keeping the gap closed requires at least 3 FTPs (two to move the momentum and one to close gap). Therefore, S_0 and S_1 cannot be both codimension-0 subspaces of v_i parameter space, and the codimension-1 gap closing at Γ can only be Z_2 or non-Z_2 but not both. Since we already show that the Z_2 transition at Γ can be codimension-1, the codimension-1 gap closing at Γ should always change the Z_2 index.

We next study the non-zero PET components, starting from the $v_1 = v_2 = 0$ case. If we further set $\xi_3 = \xi_4 = \xi_5 = \xi_6 = 0$, the electron-strain coupling $h_{p3,1}$ also has the effective inversion $\widetilde{P}$, leading to the vanishing PET. It means that ξ_1 and ξ_2 cannot contribute to the PET for $v_1 = v_2 = 0$. Indeed, the direct derivation gives the PET jump

$$\begin{aligned}\Delta\gamma_{xxx} &= -\frac{e}{\pi}\frac{\sum_{b=3}^{6} v_b\xi_b}{\sum_{b=3}^{6} v_b^2} \\ \Delta\gamma_{yyy} &= \frac{e}{\pi}\frac{v_4\xi_3 - v_3\xi_4 + v_6\xi_5 - v_5\xi_6}{\sum_{b=3}^{6} v_b^2} \ .\end{aligned} \tag{16}$$

For non-zero v_1 and v_2, the PET components can be calculated numerically for $v_3 = v_4 = v_5 = v_6 = 1eV\text{Å}$ and $\xi_1 = \xi_2 = \xi_3 = 2\xi_4 = \xi_5 = 2\xi_6 = 1eV$, showing the jump across the TQPT in Supplementary Fig. 2(a).

II. Scenario (ii): $C_3 \in \mathcal{G}_0$ and $\mathcal{T} \notin \mathcal{G}_0$

The gap closing momenta in scenario (ii) are K and K' in Fig. 1 (g) in the main text. Since these two momenta are related by $\mathcal{T}$, we only need to derive the effective model at one momentum, say K, and the other one can be obtained using $\mathcal{T}$. At K, the C_3 symmetry has three possible eigenvalues $-1, e^{\pm i\pi/3}$ due to $C_3^3 = -1$. If the gap closing is between two states with the same C_3 eigenvalues, it cannot be TQPT since the fixed gap closing momentum leads to 3 FTPs for the gap closing. (See Supplementary Note **3** C.)

There are three cases for two states with different C_3 eigenvalues: $(e^{-i\pi/3}, e^{i\pi/3})$, $(e^{i\pi/3}, -1)$, and $(-1, e^{-i\pi/3})$. The effective models in the three cases are equivalent since the representations of C_3 in these cases can be related to each other by multiplying a phase factor $e^{\pm i2\pi/3}$. Therefore, we focus on the first case, of which the effective model at K (after an appropriate unitary transformation) is given by h_+ in Supplementary Eq. (7) with

$$\begin{aligned} & v_x = v_y \equiv v,\ v_0 = 0,\ \xi_{a'',xy} = \xi_{a'',yx} = 0, \\ & \xi_{a'',xx} = \xi_{a'',yy},\ \xi_{x,xx} = -\xi_{x,yy} = -\xi_{y,xy} = -\xi_{y,yx}, \\ & \xi_{y,xx} = -\xi_{y,yy} = \xi_{x,xy} = \xi_{x,yx}\ , \end{aligned} \tag{17}$$

where $a'' = 0$ or z. Similarly, by choosing the appropriate bases at K' such that $\mathcal{T} \doteq i\sigma_y \mathcal{K}$, the effective model at K' is given h_- in Supplementary Eq. (7) with the parameter relation listed above. As a result, the gap closing between states with different C_3 eigenvalues needs 1 FTP and changes the Z_2 index since half 1BZ contains one Dirac cone (K or K'). Based on Supplementary Eq. (8), the jump of independent PET components across this TQPT (varying m from 0^- to 0^+) has the non-zero form

$$\Delta\gamma_{xxx} = -e\frac{\Delta N_+ \xi_{y,xx}}{\pi v}\ ,\ \Delta\gamma_{yyy} = e\frac{\Delta N_+ \xi_{x,yy}}{\pi v}\ , \tag{18}$$

where $\Delta N_+ = -\text{sgn}(v^2) = -1$.

III. Scenario (iii): trivial $\mathcal{G}_0$

In scenario (iii), there are six gap closing momenta, labeled as $\pm\mathbf{k}_0$, $\pm C_3\mathbf{k}_0$ and $\pm C_3^2\mathbf{k}_0$, as shown by red crosses in Fig. 1 (h) in the main text. The effective Hamiltonian at $\pm\mathbf{k}_0$ are exactly the same as Supplementary Eq. (7) since the two momenta are related by $\mathcal{T}$ and no more symmetries are involved. Therefore, the gap closing scenario needs 1 FTP, and the contribution to the PET jump from the gap closing at $\pm\mathbf{k}_0$ is the same as Supplementary Eq. (8), noted as $\Delta\gamma^{(0)}_{ijk}$. The effective models at $\pm C_3\mathbf{k}_0$ and $\pm C_3^2\mathbf{k}_0$ can be obtained from those at $\pm\mathbf{k}_0$ by C_3 and C_3^2 operations, respectively, whose electronic parts are also in the Dirac Hamitlonian form. The contracted half 1BZ then contains three Dirac cones at the gap closing and its CN must change by an odd number, indicating the change of Z_2 index. Furthermore, the contributions to the jump of PET components from the gap closing at $\pm C_3\mathbf{k}_0$ and $\pm C_3^2\mathbf{k}_0$ are $\Delta\gamma^{(1)}_{ijk} = (C_3)_{ii'}(C_3)_{jj'}(C_3)_{kk'}\Delta\gamma^{(0)}_{i'j'k'}$ and $\Delta\gamma^{(2)}_{ijk} = (C_3^2)_{ii'}(C_3^2)_{jj'}(C_3^2)_{kk'}\Delta\gamma^{(0)}_{i'j'k'}$, respectively, owing to the symmetry. As a result, the jump of independent PET components is given by $\Delta\gamma_{ijk} = \Delta\gamma^{(0)}_{ijk} + \Delta\gamma^{(1)}_{ijk} + \Delta\gamma^{(2)}_{ijk}$, which has the nonzero form

$$\begin{aligned} \Delta\gamma_{xxx} =& -e\frac{3\Delta N_+}{4\pi}\frac{2v_y\xi_{x,xy} - 2v_0\xi_{y,xy} + v_x(\xi_{y,xx} - \xi_{y,yy})}{v_x v_y} \\ \Delta\gamma_{yyy} =& e\frac{3\Delta N_+}{4\pi}\left[\frac{v_y(-\xi_{x,xx} + \xi_{x,yy}) + 2v_x\xi_{y,xy}}{v_x v_y}\right. \\ & \left. + \frac{v_0(\xi_{y,xx} - \xi_{y,yy})}{v_x v_y}\right]\ . \end{aligned} \tag{19}$$

D. PG $p31m$ and PG $p3m1$

Both PGs $p31m$ and $p3m1$ are generated by the lattice translation, the three-fold rotation C_3, and a mirror symmetry which we choose to be m_x without loss of generality. The difference between the two PGs lies on the direction of the mirror line relative to the primitive lattice vector: the mirror line is parallel or perpendicular to one primitive lattice

vector for $p31m$ or $p3m1$, respectively. C_3 and m_x span the point group C_{3v}, which leads to

$$\begin{aligned}\gamma_{xxx} = \gamma_{xyy} = \gamma_{yxy} = \gamma_{yyx} = 0 \\ \gamma_{xyx} = \gamma_{xxy} = \gamma_{yxx} = -\gamma_{yyy}\end{aligned} \tag{20}$$

for the PET, and thus γ_{yyy} serves as the only independent symmetry-allowed PET component. We classify the gap closing scenarios into 4 types according to $\mathcal{G}_0$: (i) $\mathcal{G}_0$ contains $\mathcal{T}$, (ii) $\mathcal{G}_0$ contains at least one of the three mirror symmetry operations in C_{3v} (again labeled as $\mathcal{U} = m_x$, $C_3 m_x$, or $C_3^2 m_x$) but no $\mathcal{T}$, (iii) $\mathcal{G}_0$ contains the $\mathcal{UT}$ but no $\mathcal{T}$, and (iv) $\mathcal{G}_0$ is trivial. As summarized in Tab. 1 in the main text, all gap closing cases between gapped states with 1 FTP change either Z_2 index or the VCN, and lead to the jump of symmetry-allowed PET components.

I. Scenario (i): TRIM

Similar as Supplementary Note **2** C **I** for PG $p3$, there are four inequivalent TRIM: the Γ point and three M points. Although $\mathcal{G}_0$ at the M point now contains $\mathcal{U}$, the gap closing still requires 3 FTPs same as the corresponding case in Supplementary Note **2** B **I**, which cannot be a TQPT.

When the gap closes at Γ point (Fig. 1 (i-j) in the main text), the generators of $\mathcal{G}_0$ besides the lattice translation are C_3, m_x and $\mathcal{T}$, and there are still two types of Kramers pairs characterized by the C_3 eigenvalues as those in Supplementary Note **2** C **I**. Owing to the extra mirror symmetry here, the number of FTPs for the gap closing between the same type of Kramers pairs becomes 2 for $(e^{-\mathrm{i}\pi/3}, e^{\mathrm{i}\pi/3})$ type and 3 for $(-1,-1)$ type as discussed in Supplementary Note **3** D. Therefore, we still only need to consider the gap closing between different types of Kramers pairs. For the convenience of the later material discussion, we choose the bases as $(e^{-\mathrm{i}\pi/3}, -1, e^{\mathrm{i}\pi/3}, -1)$. One can always choose the TR symmetry and mirror symmetry to be represented as $\mathcal{T} \doteq -\mathrm{i}\sigma_y\tau_0\mathcal{K}$ and $m_x \doteq -\mathrm{i}\sigma_x\tau_0$. In this case, the effective Hamiltonian can be derived by imposing the m_x on Supplementary Eq. (13), leading to

$$v_1 = v_4 = v_5 = \xi_3 = \xi_6 = 0 \ . \tag{21}$$

The form of the Hamiltonian then reads

$$\begin{aligned} h_{p3m}(\mathbf{k}, u_{ij}) &= E_0 + \xi_1 u^2 \\ &+ \begin{pmatrix} m & v_3 k_+ & \mathrm{i}v_2 k_- & -\mathrm{i}v_6 k_+ \\ v_3 k_- & -m & -\mathrm{i}v_6 k_+ & 0 \\ -\mathrm{i}v_2 k_+ & \mathrm{i}v_6 k_- & m & -v_3 k_- \\ \mathrm{i}v_6 k_- & 0 & -v_3 k_+ & -m \end{pmatrix} \\ &+ \begin{pmatrix} \xi_2 u^2 & \xi_4 u_- & 0 & \mathrm{i}\xi_5 u_- \\ \xi_4 u_+ & -\xi_2 u^2 & -\mathrm{i}\xi_5 u_- & 0 \\ 0 & \mathrm{i}\xi_5 u_+ & \xi_2 u^2 & \xi_4 u_+ \\ -\mathrm{i}\xi_5 u_+ & 0 & \xi_4 u_- & -\xi_2 u^2 \end{pmatrix} , \end{aligned} \tag{22}$$

where $u^2 = u_{xx} + u_{yy}$, $u_\pm = u_{xx} - u_{yy} \pm \mathrm{i}(u_{xy} + u_{yx})$.

The above Hamiltonian shows that the gap closing at Γ needs only 1 FTP, which is m. As discussed in Supplementary Note **3** D, this gap closing cannot drive a gapped phase into a mirror-protected gapless phase, and therefore can separate two gapped states. Similar to the discussion in Supplementary Note **2** C **I**, the gap closing changes the Z_2 index when tuning m from 0^- to 0^+, indicating a TQPT. When $v_2 = 0$, an analytical expression for the jump of independent PET component can be obtained from Supplementary Eq. (16) and Supplementary Eq. (21), which reads

$$\Delta\gamma_{yyy} = \frac{e}{\pi}\frac{-v_3\xi_4 + v_6\xi_5}{v_3^2 + v_6^2} \ . \tag{23}$$

With parameter values $v_3 = v_6 = 1eV\text{Å}$ and $\xi_1 = \xi_2 = 2\xi_4 = \xi_5 = 1eV$, the numerical results (Supplementary Fig. **2**(b)) for non-zero v_2 still show a PET jump across TQPT.

II. Scenario (ii): $\mathcal{U} \in \mathcal{G}_0$ and $\mathcal{T} \notin \mathcal{G}_0$

Scenario (ii) can be further divided into two classes depending on whether $\mathcal{G}_0$ contains C_3. When $\mathcal{G}_0$ does not contain C_3, the gap closing either requries more than 1 FTP or drives the system into a mirror-protected gapless phase with 0 codimension, similar to Supplementary Note **2** B **II**.

Only when the gap closes at K, K' for $p31m$, $\mathcal{G}_0$ contains C_3. In this case, $\mathcal{G}_0$ contains the group C_{3v}, which has one 2D irreducible representation (IR) and two different 1D IRs when acting on the states. The gap closing between the states furnishing the same IR requires 3 FTPs, similar to the case for two states with the same C_3 eigenvalue in Supplementary Note **2** C **II**. If the gap closes between the doubly degenerate states furnishing the 2D IR and a state furnishing a 1D IR, the system with a fixed carrier density cannot be insulating on both sides of the gap closing because the number of occupied bands is changed. If the gap closes between two states that furnish different 1D IRs, the mirror-protected gapless phase must exist on one side of the gap closing as the two states must have opposite mirror eigenvalues. Therefore, there is no direct TQPT between the insulating phases in scenario (ii).

III. Scenario (iii): $\mathcal{UT} \in \mathcal{G}_0$ and $\mathcal{T} \notin \mathcal{G}_0$

In scenario (iii), the gap closing cases are again divided into two different classes depending on whether $\mathcal{G}_0$ has C_3. We first discuss the class without C_3, which happens for the gap closing at $\mathcal{UT}$ invariant momenta except K, K' for $p3m1$. As shown in Fig. 1 (k-m) in the main text, the total number of inequivalent gap closing momenta is six, including $\pm\mathbf{k}_0$, $\pm C_3\mathbf{k}_0$, and $\pm C_3^2\mathbf{k}_0$. Without loss of generality, we choose $\mathbf{k}_0$ such that $-m_x\mathbf{k}_0$ is equivalent to $\mathbf{k}_0$. Then, the effective models at $\pm\mathbf{k}_0$ are the same as the corresponding models in Supplementary Note **2** B **III**, *i.e.* Supplementary Eq. (7) with the parameter relation Supplementary Eq. (9), indicating 1 FTP for the gap closing. Since the effective models at $\pm C_3\mathbf{k}_0$ and $\pm C_3^2\mathbf{k}_0$ are related to those at $\pm\mathbf{k}_0$ by C_3 and C_3^2 operations similar to Supplementary Note **2** C **III**, the jump of PET components can be derived by substituting Supplementary Eq. (9) into Supplementary Eq. (19), resulting in

$$\Delta\gamma_{yyy} = e\frac{3\Delta N_+}{4\pi}\frac{v_y(-\xi_{x,xx}+\xi_{x,yy})+2v_x\xi_{y,xy}}{v_x v_y} \ . \tag{24}$$

Moreover, since three Dirac cones exist in half 1BZ when the gap closes, the Z_2 index changes at the gap closing, making it a TQPT.

The class that $\mathcal{G}_0$ includes C_3 can only happen when the gap closes at K and K' for PG $p3m1$, as shown in Fig. 1 (m) in the main text. We can choose C_3 and $m_x\mathcal{T}$ as the generators of $\mathcal{G}_0$ besides the lattice translation. Similar to Supplementary Note **2** C **II**, we first study K and derive the model at K' by choosing the right bases such that $\mathcal{T} \doteq \mathrm{i}\sigma_y\mathcal{K}$. The states at K can be labeled by C_3 eigenvalues, -1 and $e^{\pm\mathrm{i}\pi/3}$ given by $C_3^3 = -1$. Since $(m_x\mathcal{T})^2 = 1$ and $C_3 m_x\mathcal{T} = m_x\mathcal{T}C_3^{-1}$, the gap closing typically happens between two non-degenerate states, labeled by the C_3 eigenvalues as (λ_1, λ_2), and we can always choose $m_x\mathcal{T} \doteq \mathcal{K}$. The $\lambda_1 = \lambda_2$ case cannot correspond to TQPT since 2 FTPs are needed for the gap closing as discussed in Supplementary Note **3** D, while the $\lambda_1 \neq \lambda_2$ case requires only one FTP for the gap closing similar to Supplementary Note **2** C **II**. Since the matrix representations of C_3 and $m_x\mathcal{T}$ are equivalent for the three choices $(\lambda_1, \lambda_2) = (e^{-\mathrm{i}\pi/3}, e^{\mathrm{i}\pi/3})$, $(e^{\mathrm{i}\pi/3}, -1)$, and $(-1, e^{-\mathrm{i}\pi/3})$, they have the same effective models and we only consider the first choice. With all the above conventions and simplifications, the effective models at K and K' can be given by those for Supplementary Note **2** C **II** with an extra constraint $\xi_{y,yy} = 0$ brought by $m_x\mathcal{T}$. As a result, the Z_2 index does change when the gap closes, and the jump of PET components can be derived from Supplementary Eq. (18) with the above extra constraint, which reads

$$\Delta\gamma_{yyy} = -e\frac{\Delta N_+\xi_{x,xx}}{\pi v} \ . \tag{25}$$

IV. Scenario (iv): trivial $\mathcal{G}_0$

In scenario (iv), the gap closes simultaneously at twelve inequivalent momenta, namely $\pm\mathbf{k}_0$, $\pm m_x\mathbf{k}_0$, $\pm C_3\mathbf{k}_0$, $\pm C_3 m_x\mathbf{k}_0$, $\pm C_3^2\mathbf{k}_0$ and $\pm C_3^2 m_x\mathbf{k}_0$ in Fig. 1 (n-o) in the main text. The effective model around $\mathbf{k}_0$ can be chosen as h_+ in Supplementary Eq. (7), and the models around other gap closing momenta can be further obtained by the symmetry. Although this gap closing scenario only needs 1 FTP, it cannot induce any change of Z_2 index since there is an even number (six) of Dirac cones in contracted half 1BZ. However, the gap closing can change the VCN when the twelve valleys are well defined according to Supplementary Note **2** B **IV**, *e.g.* $N_{\mathbf{k}_0}$ can change by ±1, and thus is a TQPT in the sense of the locally stable topology.

We split the change of PET components for this scenario into 3 parts: $\gamma^{(0)}$ from $\pm\mathbf{k}_0$ and $\pm m_x\mathbf{k}_0$, $\gamma^{(1)}$ from $\pm C_3\mathbf{k}_0$ and $\pm C_3 m_x\mathbf{k}_0$, and $\gamma^{(2)}$ from $\pm C_3^2\mathbf{k}_0$ and $\pm C_3^2 m_x\mathbf{k}_0$. Since the contribution to $\gamma^{(0)}$ contains two Kramers pairs that are related by m_x, same as Supplementary Note **2 B IV**, $\gamma^{(0)}$ equals to Supplementary Eq. (11). C_3 symmetry then gives $\Delta\gamma^{(1)}_{ijk} = (C_3)_{ii'}(C_3)_{jj'}(C_3)_{kk'}\Delta\gamma^{(0)}_{i'j'k'}$ and $\Delta\gamma^{(2)}_{ijk} = (C_3^2)_{ii'}(C_3^2)_{jj'}(C_3^2)_{kk'}\Delta\gamma^{(0)}_{i'j'k'}$, similar to Supplementary Note **2 C III**. As the result, the total change of PET can be obtained from $\Delta\gamma = \Delta\gamma^{(0)} + \Delta\gamma^{(1)} + \Delta\gamma^{(2)}$, which is propotional to the change of the VCN of the system

$$\Delta\gamma_{yyy} = e\frac{\Delta N_{val}}{8\pi}\left[\frac{v_y(-\xi_{x,xx}+\xi_{x,yy})+2v_x\xi_{y,xy}}{v_xv_y} + \frac{v_0(\xi_{y,xx}-\xi_{y,yy})}{v_xv_y}\right] \tag{26}$$

with $\Delta N_{val} = 12\Delta N_+$.

E. 10 PGs with 2D Inversion or C_2

The PET jump cannot exist in 10 PGs that contain C_2 or inversion, including $p2$, $p2mm$, $p2mg$, $p2gg$, $c2mm$, $p4$, $p4mm$, $p4gm$, $p6$, and $p6mm$. This conclusion can be drawn from the symmetry analysis of PET. Since both C_2 and inversion transform (x,y) to $(-x,-y)$, $\gamma_{ijk} = -\gamma_{ijk}$ is required for those 10 PGs, leading to the vanishing PET. Early study[6,8] also shows that a stable gapless phase can exist in between the QSH insulator and the NI when C_2 exists. In this gapless regime, 2D gapless Dirac fermions are locally stable and can only be created or annihilated in pairs.

Supplementary Note 3. Numbers of FTPs and Effective Models for the Gap Closing

The discussion on the gap closing between two non-degenerate states has some overlap with Supplementary Ref. [2].

A. PG $p1$

This part has been studied in Supplementary Ref. [3].

I. Not TRIM

When the gap closes at $\mathbf{k}_0$ that is not a TRIM, the two-band model near the gap closing to the leading order of $\mathbf{q} = \mathbf{k} - \mathbf{k}_0$ in general takes the form

$$h(\mathbf{q}) = E_0(\mathbf{q})\sigma_0 + (q_x\mathbf{C}_x + q_y\mathbf{C}_y + \mathbf{M})\cdot\boldsymbol{\sigma}\ , \tag{27}$$

where $\mathbf{C}_i = (C_{ix}, C_{iy}, C_{iz})$, $\mathbf{M} = (M_x, M_y, M_z)$, $\boldsymbol{\sigma} = (\sigma_x, \sigma_y, \sigma_z)$, and the two bases of the above model account for the doubly degenerate band touching when the gap closes. Supplementary Eq. (27) determines the codimension for the gap closing scenario since the gap at $-\mathbf{k}_0$ is related to that of Supplementary Eq. (27) by the TR symmetry. The gap of Supplementary Eq. (27) closes if and only if $\mathbf{C}_xq_x + \mathbf{C}_yq_y + \mathbf{M} = 0$. We choose $\mathbf{C}_x \times \mathbf{C}_y \neq 0$ since it can be satisfied without finely tuning anything (or equivalently in a parameter subspace with 0 codimension). In this case, the gap closes when $\mathbf{M}$ lies in the plane spanned by two vectors $\mathbf{C}_x$ and $\mathbf{C}_y$. Therefore, the codimension for the gap closing is 1 since only the angle between the vector $\mathbf{M}$ and the $(\mathbf{C}_x, \mathbf{C}_y)$ plane needs to be tuned.

Next, we derive Eq. (5) of the main text and the electronic part of h_+ in Supplementary Eq. (7), while the model at $-\mathbf{k}_0$ can be derived by the TR symmetry and thus is not discussed here. Supplementary Eq. (27) always allows the $\mathbf{q}$-independent SU(2) transformation, *i.e.* $h(\mathbf{q}) \to U^\dagger h(\mathbf{q})U$ with $U \in$ SU(2). Such transformation only changes the bases of the Hamiltonian but does not change the direction of the momentum or the coordinate system. Since $\boldsymbol{\sigma}$ behaves as an SO(3) vector under U, every SU(2) transformation U of the Hamiltonian is equivalent to an SO(3) transformation R of the vectors $\mathbf{C}_i$ and $\mathbf{M}$, *i.e.* $\mathbf{C}_i \to R\mathbf{C}_i$ and $\mathbf{M} \to R\mathbf{M}$. Thus, by choosing appropriate U matrix,

we can first rotate $\mathbf{C}_x$ to the x direction and then $\mathbf{C}_y$ to the xy plane, resulting in $R\mathbf{C}_x = v_x\mathbf{e}_x$, $R\mathbf{C}_y = v_0\mathbf{e}_x + v_y\mathbf{e}_y$ and $R\mathbf{M} = m_1\mathbf{e}_x + m_2\mathbf{e}_y + m\mathbf{e}_z$. As a result, Supplementary Eq. (27) is transformed to

$$\begin{aligned} h(\mathbf{q}) =& E_0(\mathbf{q})\sigma_0 + (v_xq_x + v_0q_y + m_1)\sigma_x + (v_yq_y + m_2)\sigma_y \\ &+ m\sigma_z \ . \end{aligned} \tag{28}$$

Here $\mathbf{C}_x \times \mathbf{C}_y \neq 0$ gives non-zero v_x and v_y. With a shift of $\mathbf{k}_0$ by $(m_1/v_x - v_0m_2/(v_xv_y), m_2/v_y)$, the model is further simplified to the electronic part of h_+ in Supplementary Eq. (7). Finally, we define the $q_x + v_0q_y$ and q_y to be q_1 and q_2, respectively, to get Eq. (5) , which represents the most generic form of the Hamiltonian.

II. TRIM

In this part, we count the number of FTPs for the gap closing at the TRIM. Owing to the Kramers' degeneracy, every band at the TRIM is doubly degenerate, and we use the name "Kramers pair" to label the two states related by the TR symmetry. We consider the gap closing between two Kramers pairs $|1,\pm\rangle$ and $|2,\pm\rangle$, where $\mathcal{T}|i,+\rangle = -|i,-\rangle$ can always be chosen by the unitary transformation. As a result, the mass term for the effective model at the TRIM reads

$$\begin{pmatrix} m_1 & 0 & \Delta_0 + \mathrm{i}\Delta_3 & \mathrm{i}\Delta_1 + \Delta_2 \\ 0 & m_1 & \mathrm{i}\Delta_1 - \Delta_2 & \Delta_0 - \mathrm{i}\Delta_3 \\ \Delta_0 - \mathrm{i}\Delta_3 & -\mathrm{i}\Delta_1 - \Delta_2 & m_2 & 0 \\ -\mathrm{i}\Delta_1 + \Delta_2 & \Delta_0 + \mathrm{i}\Delta_3 & 0 & m_2 \end{pmatrix} \tag{29}$$

where the bases are $(|1,+\rangle, |1,-\rangle, |2,+\rangle, |2,-\rangle)$ and all parameters are real. Since the momentum is fixed at TRIM ($-\mathbf{k} = \mathbf{k} + \mathbf{G}$ with $\mathbf{G}$ a reciprocal lattice vector), none of the terms in the above equation can be canceled by shifting the momentum. Therefore, 5 FTPs are needed for the gap closing.

B. $p1m1$, $c1m1$, and $p1g1$

I. Scenario (i): TRIM

If $\mathcal{G}_0$ does not contain $\mathcal{U}$, which can occur on the edge of 1BZ for $c1m1$, the situation is the same as the TRIM in Supplementary Note **3** A, which requires 5 FTPs. When $\mathcal{G}_0$ contains $\mathcal{U}$, we should discuss the $\mathcal{U} = m_x$ case ($p1m1$ and $c1m1$) and the $\mathcal{U} = g_x$ case ($p1g1$), separately.

For $p1m1$ and $c1m1$, since $m_x^2 = -1$, two states of one Kramers pair have opposite mirror eigenvalues $\pm\mathrm{i}$, labeled by $m_x|i,\pm\rangle = \pm\mathrm{i}|i,\pm\rangle$. On the bases $(|1,+\rangle, |1,-\rangle, |2,+\rangle, |2,-\rangle)$, the effective model around the gap closing between two Kramers pairs can be given by Supplementary Eq. (29) with $\Delta_1 = \Delta_2 = 0$, since the bases with different mirror eigenvalues cannot be coupled by the mass terms. As a result, 3 FTPs are needed for such gap closing scenario.

For $p1g1$, $g_x^2 = -1$ at Γ and X and the number of FTPs for the gap closing is thus the same as the above case, which is 3. At Y and M, $g_x^2 = 1$ and two states of one Kramers pair have the same g_x eigenvalue, 1 or -1. In this case, the gap closing between two Kramers pairs with the same g_x eigenvalue needs 5 FTPs, which is the same as the TRIM scenario in Supplementary Note **3** A. On the other hand, between two Kramers pairs with opposite g_x eigenvalues, only 1 FTP needs to be tuned to close the gap at Y or M, since the off-diagonal terms ($\Delta_{0,1,2,3}$) in Supplementary Eq. (29) are all forbidden.

II. Scenario (ii): $\mathcal{U} \in \mathcal{G}_0$ but $\mathcal{T} \notin \mathcal{G}_0$

In scenario (ii), there are two gap closing momenta $\pm\mathbf{k}_0$ that are related by the TR symmetry. Therefore, we only need to consider one of them, say $\mathbf{k}_0$, to derive the number of FPTs for the gap closing. At $\mathbf{k}_0$, the states can be labeled by the eigenvalues of $\mathcal{U}$. If the gap closing between two states with the same $\mathcal{U}$ eigenvalues, the effective model can be described by Supplementary Eq. (27) with $|\mathbf{C}_x| = 0$. The gap closes if and only if $\mathbf{C}_yq_y + \mathbf{M} = 0$, realizable by making two vectors $\mathbf{M}$ and $\mathbf{C}_y$ parallel. Such realization needs 2 FTPs, *e.g.* the two components of the projection of $\mathbf{M}$ on the plane perpendicular to $\mathbf{C}_y$.

IR	Expressions
$A_1,+$	$\tau_+\sigma_0,\ \tau_-\sigma_0,\ u_{11}+u_{22}$
$A_1,-$	$\tau_+\sigma_z,\ \tau_-\sigma_x,\ \tau_-\sigma_y,\ \tau_-\sigma_z$
$E,+$	$(\tau_y\sigma_z-\mathrm{i}\tau_x\sigma_0,\tau_y\sigma_z+\mathrm{i}\tau_x\sigma_0),\ (\tau_y\sigma_x+\mathrm{i}\tau_y\sigma_y,\tau_y\sigma_x-\mathrm{i}\tau_y\sigma_y),\ (-u_{xx}+u_{yy}-\mathrm{i}(u_{xy}+u_{yx}),-u_{xx}+u_{yy}+\mathrm{i}(u_{xy}+u_{yx}))$
$E,-$	$(\tau_+(\sigma_x-\mathrm{i}\sigma_y),\tau_+(\sigma_x+\mathrm{i}\sigma_y)),\ (\tau_x\sigma_z+\mathrm{i}\tau_y\sigma_0,\tau_x\sigma_z-\mathrm{i}\tau_y\sigma_0),\ (\tau_x\sigma_x+\mathrm{i}\tau_x\sigma_y,\tau_x\sigma_x-\mathrm{i}\tau_x\sigma_y),\ (k_x-\mathrm{i}k_y,k_x+\mathrm{i}k_y)$

Supplementary Table 1. The irreducible representations (IRs) of C_3 and TR symmetries. In A_1 IR, the C_3 eigenvalue of the bases is 1 and $\pm$ are parity under TR. "$E,\pm$" label two 2D IRs, where the two components have the C_3 eigenvalues $(e^{\mathrm{i}2\pi/3},e^{-\mathrm{i}2\pi/3})$ and transform as $\pm\sigma_x\mathcal{K}$ under the TR symmetry. $\tau_\pm=(\tau_0\pm\tau_z)/2$.

When the gap closes between two states with different $\mathcal{U}$ eigenvalues, the effective model along the $\mathcal{U}$-invariant line ($q_x=0$) reads $h(\mathbf{q})=E_0(q_y)+(m_0+Cq_y+B_0q_y^2)\sigma_z$, which, by shifting the $k_{0,y}$, can be simplified to $h(\mathbf{q})=E_0(q_y)+(m+Bq_y^2)\sigma_z$. The gap for this Hamiltonian keeps closing when $mB\le 0$, indicating a stable gapless phase protected by $\mathcal{U}$ with 0 codimension.

III. Scenario (iii): $\mathcal{UT}\in\mathcal{G}_0$ but $\mathcal{T}\notin\mathcal{G}_0$

In this scenario, we here only consider the $(\mathcal{UT})^2=-1$ case, where each band at the gap closing momentum is doubly degenerate. We can define the $\mathcal{UT}$ pair as the two degenerate states related by $\mathcal{UT}$, in analog to the Kramers pair defined in Supplementary Note **3** A. Similar as Supplementary Eq. (29), there are 5 mass terms for the gap closing between two $\mathcal{UT}$ pairs. However, the case here is different from the TRIM scenario in Supplementary Note **3** A, since q_x does not change under $\mathcal{UT}$ and thus the corresponding terms have the same form as the mass terms in Supplementary Eq. (29). One of the five mass terms can then be canceled by shifting $k_{0,x}$, resulting in 4 FTPs for the gap closing.

C. p_3

I. Scenario (i):TRIM

We first discuss the gap closing at Γ between two Kramers pairs of the same type. If the bases have C_3 eigenvalues $(e^{-\mathrm{i}\pi/3},e^{\mathrm{i}\pi/3},e^{-\mathrm{i}\pi/3},e^{\mathrm{i}\pi/3})$, the mass term of the effective model is given by Supplementary Eq. (29) with $\Delta_1=\Delta_2=0$ since the bases with different C_3 eigenvalues cannot be coupled, resulting in 3 FTPs for the gap closing. If the bases have C_3 eigenvalues $(-1,-1,-1,-1)$, the effective model equals to Supplementary Eq. (29) that has 5 FTPs for the gap closing.

Now we discuss the construction of the effective model for the bases $(e^{-\mathrm{i}\pi/3},e^{\mathrm{i}\pi/3},-1,-1)$. The form of the effective model, Supplementary Eq. (13), is given by the tensor product of the bases in the same IR listed in Supplementary Tab. **1**. Note that the matrix representation and the bases for the E IR are not Hermitian. It means given two copies of $(E,+)$ or $(E,-)$ IR, say $(\tau_+(\sigma_x-\mathrm{i}\sigma_y),\tau_+(\sigma_x+\mathrm{i}\sigma_y))$ and $(k_x-\mathrm{i}k_y,k_x+\mathrm{i}k_y)$ furnishing $(E,-)$ IR, the coefficients used for the tensor product can be complex, *e.g.* $c[\tau_+(\sigma_x-\mathrm{i}\sigma_y)][k_x-\mathrm{i}k_y]^*+c^*[\tau_+(\sigma_x+\mathrm{i}\sigma_y)][k_x+\mathrm{i}k_y]^*$ with complex c.

II. Scenario (ii): $C_3\in\mathcal{G}_0$ and $\mathcal{T}\notin\mathcal{G}_0$

Here we consider the gap closing between two states with the same C_3 eigenvalues at K or K'. In general, the mass terms at one gap closing momentum are $m_x\sigma_x+m_y\sigma_y+m_z\sigma_z$. Since the gap closing momentum is fixed, none of the three mass terms can be canceled by shifting the momentum, and hence there are 3 FTPs for the gap closing.

D. $p31m$ and $p3m1$

I. Scenario (i): TRIM

When the two Kramers pairs carry C_3 eigenvalues as $(e^{-\mathrm{i}\pi/3}, e^{\mathrm{i}\pi/3}, e^{-\mathrm{i}\pi/3}, e^{\mathrm{i}\pi/3})$, the effective model equals to Supplementary Eq. (29) with $\Delta_1 = \Delta_2 = 0$ before considering m_x, similar to the correspond case in Supplementary Note 3 C. As $m_x \doteq -\mathrm{i}\sigma_x$ for each Kramers pair, the Δ_3 is also forbidden, resulting in 2 FTPs for the gap closing. On the other hand, if C_3 eigenvalues are all -1, the effective model equals to Supplementary Eq. (29) before considering m_x, similar to the correspond case in Supplementary Note 3 C, and including m_x makes $\Delta_2 = \Delta_3 = 0$, leading to 3 FTPs for the gap closing.

The construction of the effective model for the bases $(e^{-\mathrm{i}\pi/3}, -1, e^{\mathrm{i}\pi/3}, -1)$ is the same as that for the (111) HgTe/CdTe quantum well, which is discussed in Supplementary Note 5. Next we show that the gap closing at Γ in this case cannot drive a gapped system to the mirror protected gapless phase. Since the three mirror lines are related by the C_3 symmetry, we only need to consider one of them, say $k_x = 0$ that is invariant under m_x. The eigenvalues along this line read

$$E_{\alpha\beta} = E_0 + \alpha\frac{k_y v_2}{2} + \beta\sqrt{(m+\alpha\frac{v_2 k_y}{2})^2 + k_y^2(v_3^2+v_6^2)} \tag{30}$$

with α, β take $\pm$. $E_{\pm\beta}$ bands cross at Γ and belong to the same set of connected bands. The mirror eigenvalue of the $E_{\alpha\beta}$ band is $-\alpha\mathrm{i}$, and then the mirror protected gapless phase happens when E_{++} crosses with E_{--} or E_{+-} crosses with E_{-+}. Both band crossings require the same condition

$$|v_2 k_y| = \sum_\alpha \sqrt{(m+\alpha\frac{v_2 k_y}{2})^2 + k_y^2(v_3^2+v_6^2)}, \tag{31}$$

since they are related by the TR symmetry. However, the above equation has no solution when $m \neq 0$ and $v_3^2+v_6^2 \neq 0$. It can be seen from the inequality $\sqrt{(a+b)^2+c^2} + \sqrt{(a-b)^2+c^2} > 2|b|$, which holds unless $c = 0$ and $|a| \leq |b|$. Therefore, without finely tuning more parameters to realize $v_3^2 + v_6^2 = 0$, a gapped system remains when the sign of m flips.

II. Scenario (iii): $\mathcal{UT} \in \mathcal{G}_0$ and $\mathcal{T} \notin \mathcal{G}_0$

Here we discuss the case when the gap closes at K and K' for PG $p3m1$ and between two states with the same C_3 eigenvalues. Before considering $m_x\mathcal{T}$, the mass terms at K are $m_x\sigma_x + m_y\sigma_y + m_z\sigma_z$ since C_3 does not provide any constraints and the fixed gap closing momentum cannot be shifted to cancel any of them. Since $m_x\mathcal{T}$ can be chosen as $\sigma_0\mathcal{K}$, m_y is forbideen and the remaining two mass terms serve as the 2 FTPs for the gap closing.

Supplementary Note 4. VCN in Tight-Binding Model

In this section, we discuss the quantization and physical meaning of the VCN change in a tight-binding (TB) model with $p1m1$. We consider a square lattice and each unit cell only contains one atom. Without loss of generality, we set the lattice constant to 1, and choose the mirror symmetry as m_y. On each atom, we include a spinful s and a spinful p_y orbitals, meaning that the bases can be labeled as $|\mathbf{R}, \alpha, s\rangle$ with $\mathbf{R}$ the lattice vector, $\alpha = s, p_y$ for orbital, and $s = \uparrow, \downarrow$ for spin. The bases with specific Bloch momentum can be obtained by the following Fourier transformation

$$|\mathbf{k}, \alpha, s\rangle = \frac{1}{\sqrt{N}}\sum_{\mathbf{k}} e^{\mathrm{i}\mathbf{k}\cdot\mathbf{R}}|\mathbf{R}, \alpha, s\rangle\ . \tag{32}$$

Then, the representations of the symmetries read

$$\begin{aligned} m_y|\mathbf{k}, \alpha, s\rangle &= |m_y\mathbf{k}, \alpha', s'\rangle(\tau_z)_{\alpha'\alpha}(-\mathrm{i}\sigma_y)_{s's} \\ \mathcal{T}|\mathbf{k}, \alpha, s\rangle &= |-\mathbf{k}, \alpha, s'\rangle(\mathrm{i}\sigma_y)_{s's}\ , \end{aligned} \tag{33}$$

where τ and σ are Pauli matrices for orbital and spin.

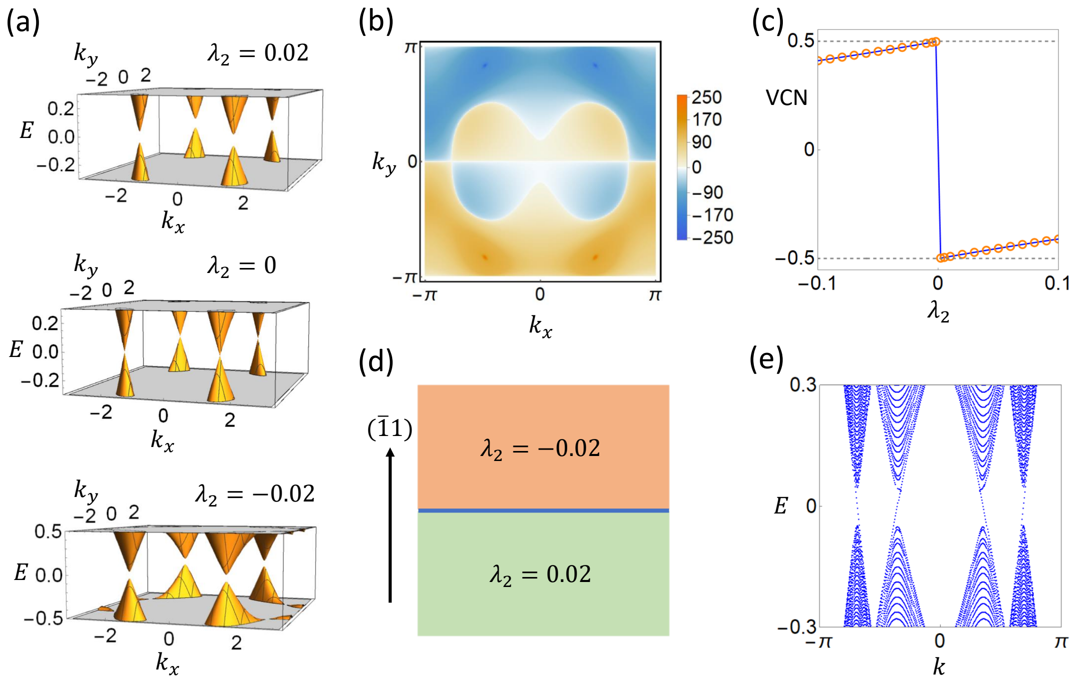

Supplementary Fig. 4. (a) shows the energy dispersion of the TB model Supplementary Eq. (34) for $\lambda_2 = 0.02, 0, -0.02$. The gap is zero for $\lambda_2 = 0$. (b) shows the distribution of the Berry curvature for $\lambda_2 = 0.02$. The peaks indicate the locations of valleys. (c) shows the integration of Berry curvature divided by 2π over the $k_{x,y} > 0$ quarter of 1BZ. The orange circles are the data points, based on which the blue line is plotted. (d) is a domain wall structure along $(\bar{1}1)$ direction. The lower and upper parts are given by the TB models for $\lambda_2 = -0.02$ and $\lambda_2 = 0.02$, respectively. (e) plots the energy dispersion of domain-wall modes (modes with considerable probability near the interface in (d)). Here we choose the number of unit cells along $(\bar{1}1)$ to be 70 for each part of the domain wall.

With on-site terms and nearest-neighbor hopping terms, we choose the following symmetry-allowed expression for the Hamiltonian

$$h(\mathbf{k}) = d_1\tau_z\sigma_0 + d_2\tau_y\sigma_z + d_3\tau_x\sigma_z + d_4\tau_y\sigma_0 \ , \tag{34}$$

where

$$\begin{aligned} d_1 &= m + 2t_1\cos(k_x) + 2t_2\cos(k_y) \\ d_2 &= \lambda_1 \\ d_3 &= 2\lambda_2\sin(k_x) \\ d_4 &= 2t_3\sin(k_y) \ . \end{aligned} \tag{35}$$

The eigenvalues of $h(\mathbf{k})$ read $\pm\sqrt{d_1^2 + d_3^2 + (d_2 \pm d_4)^2}$. For concreteness, we choose $t_1 = t_2 = t_3 = \lambda_1 = 1/2$ and $m = 4/5$ and assume the model is half-filled (two occupied bands). In this case, the gap closes only when we tune λ_2 to zero, as shown in Supplementary Fig. 4(a), and the gap closing points sit at $\mathbf{k} = (\pm\arccos(\frac{-8+5\sqrt{3}}{10}), \pm 5\pi/6) \approx (\pm 1.50, \pm 2.62)$, belonging to the VCN scenario (iv) for $p1m1$. When the gap is small but nonzero, the positions of valleys can be determined numerically by locating the peaks of Berry curvature (Supplementary Fig. 4(b)), which are close to the gap closing points.

Finally, based on the TB model Supplementary Eq. (34), we discuss the quantization and physical consequence of the VCN change across the $\lambda_2 = 0$ gap closing. Without loss of generality, we take the valley in the $k_{x,y} > 0$ quarter of 1BZ as an example to discuss the quantization. The VCN of this valley can be calculated by integrating the Berry curvature over the $k_{x,y} > 0$ quarter of 1BZ. As shown in Supplementary Fig. 4(c), although VCN is not quantized on any side of the gap closing, the change of VCN across the gap closing is an integer, consistent with the effective-model analysis in the main text. According to Supplementary Ref. [9], one physical consequence of the quantized VCN change is the gapless domain-wall mode in a domain wall structure that consists of the two different gapped phases separated by the gap closing, like Supplementary Fig. 4(d). As shown in Supplementary Fig. 4(e), the VCN change for each valley matches the number of gapless domain-wall modes around that valley.

Supplementary Note 5. (111) HgTe/CdTe Quantum Well

In this section, we provide more details on the analysis of the HgTe QW. Before going into details, we first introduce some basic properties of the QW. Both HgTe and CdTe have the standard zinc-blende structure, similar to most II-VI or III-V compound semiconductors. The crystallographic space group of both compounds is $F\bar{4}3m$ (space group No. 216). In the QW, HgTe serves as a well while $Hg_{1-x}Cd_xTe$ serves as the barrier. Similar to early experimental and theoretical studies[10–14], we use $x = 0.7$.

A. d-induced PET jump for $\mathcal{E} = 0$

To describe the TPQT, we project the 6-band Kane model onto the bases $(|E_1,+\rangle, |H_1,+\rangle, |E_1,-\rangle, |H_1,-\rangle)$ via second order perturbation[13] and get the following 4-band model

$$h^{(0)}_{eff}(\mathcal{E}=0) = E_0 + B_0 k_{\shortparallel}^2 + \begin{pmatrix} Bk_{\shortparallel}^2 + m & A_1 k_+ & 0 & -\mathrm{i}A_2 k_+ \\ A_1 k_- & -Bk_{\shortparallel}^2 - m & -\mathrm{i}A_2 k_+ & 0 \\ 0 & \mathrm{i}A_2 k_- & Bk_{\shortparallel}^2 + m & -A_1 k_- \\ \mathrm{i}A_2 k_- & 0 & -A_1 k_+ & -Bk_{\shortparallel}^2 - m \end{pmatrix}, \tag{36}$$

where the values of the parameters are listed Supplementary Tab. 3, $k_{\shortparallel}^2 = k_1^2 + k_2^2$, $k_\pm = k_1 \pm \mathrm{i}k_2$, and k_1 and k_2 are the momenta along $(1,-1,0)$ and $(1,1,-2)$, respectively. Compared to the celebrated Bernevig-Hughes-Zhang model[10], we have an additional k-linear term A_2 due to the reduction of the full rotational symmetry to C_3 rotation symmetry. In the Supplementary Eq. (36), the TQPT shown in Fig. 2 (a) of the main text occurs at $m = 0$. To show the jump of the symmetry-allowed PET components at the gap closing, we need to introduce the electron-strain coupling $h^{(1)}_{eff}$ based on the symmetry:

$$h^{(1)}_{eff} = \xi_1 u^2 + \begin{pmatrix} \xi_2 u^2 & \xi_3 u_- & 0 & -\mathrm{i}\xi_4 u_- \\ \xi_3 u_+ & -\xi_2 u^2 & \mathrm{i}\xi_4 u_- & 0 \\ 0 & -\mathrm{i}\xi_4 u_+ & \xi_2 u^2 & \xi_3 u_+ \\ \mathrm{i}\xi_4 u_+ & 0 & \xi_3 u_- & -\xi_2 u^2 \end{pmatrix}, \tag{37}$$

where $u^2 = u_{11} + u_{22}$ and $u_\pm = u_{11} - u_{22} \pm \mathrm{i}(u_{12} + u_{21})$. This electron-strain coupling is in the most general symmetry-allowed form to the leading order of u_{ij}, which definitely includes the IB terms, ξ_3 and ξ_4. With Supplementary Eq. (36) and Supplementary Eq. (37), the independent PET component γ_{222} can be derived analytically as

$$\gamma_{222} = -e\frac{\mathrm{sgn}(m)(A_1\xi_3 + A_2\xi_4)}{2\pi\,(A_1^2 + A_2^2)} \frac{(A_1^2 + A_2^2 - 2\,|Bm| + 2Bm)}{(A_1^2 + A_2^2 + 4Bm)}, \tag{38}$$

resulting in the PET jump as

$$\Delta\gamma_{222} = -e\frac{A_1\xi_3 + A_2\xi_4}{\pi\,(A_1^2 + A_2^2)}. \tag{39}$$

Based on Supplementary Eq. (38) and $\xi_{1,2,3,4} = 1\mathrm{eV}$ (comparable to those in Supplementary Ref. [5]), we plot the γ_{222} of the function of the width in Fig. 2 (b) of the main text, which shows a jump around $d = 65$Å.

B. $\mathcal{E}$-induced PET jump for fixed d

After including the electric field, we project the modified Kane model onto the bases $(|E_1,+\rangle, |H_1,+\rangle, |E_1,-\rangle, |H_1,-\rangle)$ via second order perturbation and get the following 4-band model

$$h_{eff}^{(0)} = E_0 + B_0 k_{\shortparallel}^2 + \begin{pmatrix} Bk_{\shortparallel}^2 + m & A_1 k_+ + D_1 k_-^2 & -\mathrm{i} D_3 k_- & -\mathrm{i} A_2 k_+ - \mathrm{i} D_2 k_-^2 \\ A_1 k_- + D_1 k_+^2 & -Bk_{\shortparallel}^2 - m & -\mathrm{i} A_2 k_+ + \mathrm{i} D_2 k_-^2 & 0 \\ \mathrm{i} D_3 k_+ & \mathrm{i} A_2 k_- - \mathrm{i} D_2 k_+^2 & Bk_{\shortparallel}^2 + m & D_1 k_+^2 - A_1 k_- \\ \mathrm{i} A_2 k_- + \mathrm{i} D_2 k_+^2 & 0 & D_1 k_-^2 - A_1 k_+ & -Bk_{\shortparallel}^2 - m \end{pmatrix}. \quad (40)$$

Compared with Supplementary Eq. (36), the above Hamiltonian has three extra IB terms $D_{1,2,3}$ brought by the electric field. In fact, it is now in the most general symmetry-allowed form up to the second order of the momentum for the HgTe/CdTe QW along the (111) direction. In addition, the parameter m (mass term) can also be controlled by electric field. In the contrast to (001) QW, the constant (k-independent) IB terms in Supplementary Ref. [11] are forbidden in Supplementary Eq. (40) by the C_3 symmetry. The $\mathcal{E}$ dependence of the parameters are shown in Supplementary Tab. **4** for $d = 62$Å. Since Supplementary Eq. (37) is in the most general form, the electron-strain coupling for $\mathcal{E} \neq 0$ still keeps the form of Supplementary Eq. (37). With Supplementary Eq. (40), Supplementary Eq. (37), the parameter expression, and $\xi_{1,2,3,4} = 1$eV comparable as those in Supplementary Ref. [5], the PET jump can be calculated.

C. Projection of the Kane Model

With bases $(|\Gamma_6, \frac{1}{2}\rangle, |\Gamma_6, -\frac{1}{2}\rangle, |\Gamma_8, \frac{3}{2}\rangle, |\Gamma_8, \frac{1}{2}\rangle, |\Gamma_8, -\frac{1}{2}\rangle, |\Gamma_8, -\frac{3}{2}\rangle)$, the 6-band Kane model that we use for the (111) quantum well without the electric field reads

$$h_{Kane}(\mathbf{k}) = \begin{pmatrix} h_{\Gamma_6}(\mathbf{k}) & T(\mathbf{k}) \\ T^\dagger(\mathbf{k}) & h_{\Gamma_8}(\mathbf{k}) \end{pmatrix}, \quad (41)$$

where $\mathbf{k} = (k_1, k_2, k_3)$ with $k_3 = -\mathrm{i}\partial_{x_3}$, $h_{\Gamma_6}(\mathbf{k}) = \left(E_c + \frac{\hbar^2}{2m_0}\left[(2F+1)(k_1^2 + k_2^2) + k_3(2F+1)k_3\right]\right)\sigma_0$,

$$T(\mathbf{k}) = \begin{pmatrix} -\frac{1}{\sqrt{2}} k_+ P & \sqrt{\frac{2}{3}} k_3 P & \frac{1}{\sqrt{6}} k_- P & 0 \\ 0 & -\frac{1}{\sqrt{6}} k_+ P & \sqrt{\frac{2}{3}} k_3 P & \frac{1}{\sqrt{2}} k_- P \end{pmatrix}, \quad (42)$$

$$h_{\Gamma_8}(\mathbf{k}) = \begin{pmatrix} U+V & W & \widetilde{W} & 0 \\ W^\dagger & U-V & 0 & \widetilde{W} \\ \widetilde{W}^\dagger & 0 & U-V & -W \\ 0 & \widetilde{W}^\dagger & -W^\dagger & U+V \end{pmatrix}, \quad (43)$$

$U = E_v - \frac{\hbar^2}{2m_0}[(k_1^2+k_2^2)\gamma_1 + k_3\gamma_1 k_3]$, $V = \frac{\hbar^2}{2m_0}[-(k_1^2+k_2^2)\gamma_3 + 2k_3\gamma_3 k_3]$, $W = \frac{1}{\sqrt{3}}\frac{\hbar^2}{2m_0}[-\mathrm{i}\sqrt{2}k_+^2(\gamma_2-\gamma_3) + k_-\{k_3, 2\gamma_2+\gamma_3\}]$, $\widetilde{W} = \frac{1}{\sqrt{3}}\frac{\hbar^2}{2m_0}[k_-^2(\gamma_2 + 2\gamma_3) - \mathrm{i}\sqrt{2}k_+\{k_3, \gamma_2 - \gamma_3\}]$, m_0 is the mass of the electron, and the IB effect is neglected. The electric field can be included by adding

$$V_e = -e\mathcal{E}x_3 \mathbb{1}_6 \quad (44)$$

to Supplementary Eq. (41).

Due to the spatial dependence of the parameters, we require the anti-commutation form of some k_3-dependent terms, such as $\{k_3, \gamma_2\}$, to keep the Hamiltonian hermitian[15]. The quantum well considered has the structure $Hg_{0.3}Cd_{0.7}Te/HgTe/Hg_{0.3}Cd_{0.7}Te$, leading to the x_3 dependence of parameters $X = E_{v,c}, F, \gamma_{1,2,3}$ as

$$X = \begin{cases} X^A, & |x_3| < \frac{d}{2} \\ X^B, & |x_3| > \frac{d}{2} \end{cases}. \quad (45)$$

The numerical values of the parameters in Supplementary Eq. (41) are listed in Supplementary Tab. **2**.

E_v^A	E_c^A	F^A	γ_1^A	γ_2^A	γ_3^A	P
0	-0.303	0	4.1	0.5	1.3	8.47
E_v^B	E_c^B	F^B	γ_1^B	γ_2^B	γ_3^B	
-0.399	0.607	-0.063	2.26	-0.046	0.411	

Supplementary Table 2. Values of parameters in Supplementary Eq. (41) for $Hg_{0.3}Cd_{0.7}Te/HgTe/Hg_{0.3}Cd_{0.7}Te$ quantum well. The unites of E_v, E_c are eV, the unit of P is eVÅ, and other parameters are dimensionless[14,16].

d/Å	m_0/eV	B_0/(eV Å^2)	m/eV	B/(eV Å^2)	A_1/(eV Å)	A_2/(eV Å)
60.00	-0.006700	39.88	0.005600	60.45	3.595	0.1248
61.00	-0.007570	40.90	0.004370	61.46	3.582	0.1237
62.00	-0.008400	41.94	0.003200	62.51	3.569	0.1225
63.00	-0.009240	42.99	0.002040	63.56	3.555	0.1214
64.00	-0.01009	44.08	0.0008850	64.65	3.540	0.1204
65.00	-0.01084	45.14	-0.0001650	65.71	3.527	0.1193
66.00	-0.01159	46.25	-0.001210	66.82	3.514	0.1182
67.00	-0.01235	47.41	-0.002250	67.98	3.500	0.1172
68.00	-0.01307	48.54	-0.003230	69.12	3.486	0.1162
69.00	-0.01374	49.75	-0.004160	70.33	3.473	0.1152
70.00	-0.01442	50.98	-0.005080	71.56	3.459	0.1143

Supplementary Table 3. Parameter values for Supplementary Eq. (36) at various widths d.

The effective models are derived according to Supplementary Ref. [10 and 13]. We first numerically obtain the wavefunctions of E1, H1, LH1, HH2, and HH3 bands at $k_1 = k_2 = \mathcal{E} = 0$, and project the remaining terms to the bases to get a 10×10 Hamiltonian. Then, we project the 10×10 Hamiltonian to the E1 and H1 bands with second order perturbation to get Supplementary Eq. (36) and Supplementary Eq. (40). Keeping terms up to k^2 and $\mathcal{E}^2$ order, the values of the parameters are listed in Supplementary Tab. **3** and Supplementary Tab. **4**.

D. Construction of the Hamiltonian based on symmetry

As discussed in the main text, the symmetry group of interest is generated by the three-fold rotation C_3 along (111), and the mirror $m_{1\bar{1}0}$ perpendicular to $(1, \bar{1}, 0)$ and the TR operation $\mathcal{T}$. With the bases $(|E_1, +\rangle, |H_1, +\rangle, |E_1, -\rangle, |H_1, -\rangle)$,

m_0/eV	B_0/(eV Å^2)	m/eV
4182 $(-e\mathcal{E}\text{Å}/eV)^2$−0.008400	544800 $(-e\mathcal{E}\text{Å}/eV)^2$+41.94	0.003200 -17.21 $(-e\mathcal{E}\text{Å}/eV)^2$
B/(eV Å^2)	A_1/(eV Å)	A_2/(eV Å)
534600 $(-e\mathcal{E}\text{Å}/eV)^2$+62.51	67320 $(-e\mathcal{E}\text{Å}/eV)^2$+3.569	293.0 $(-e\mathcal{E}\text{Å}/eV)^2$+0.1225
D_1/(eV Å^2)	D_2/(eV Å^2)	D_3/(eV Å)
724.7 $(-e\mathcal{E}\text{Å}/eV)$	-1947 $(-e\mathcal{E}\text{Å}/eV)$	-1196 $(-e\mathcal{E}\text{Å}/eV)$

Supplementary Table 4. Parameter values for Supplementary Eq. (40) for $d = 62$Å.

IR	Expressions
$A_1, +$	$\sigma_0\tau_0$, $\sigma_0\tau_z$, $k_1^2 + k_2^2$, $u_{11} + u_{22}$
$A_1, -$	$\sigma_x\tau_-$
$A_2, +$	
$A_2, -$	$\sigma_y\tau_-$, $\sigma_z\tau_0$, $\sigma_z\tau_z$
$E, +$	$(\sigma_z\tau_y, \sigma_0\tau_x)$, $(\sigma_y\tau_y, \sigma_x\tau_y)$, $(2k_1k_2, k_1^2 - k_2^2)$, $(u_{12} + u_{21}, u_{11} - u_{22})$
$E, -$	$(\sigma_z\tau_x, -\sigma_0\tau_y)$,$(\sigma_y\tau_x, \sigma_x\tau_x)$,$(\sigma_y\tau_+, -\sigma_x\tau_+)$, (k_1, k_2)

Supplementary Table 5. The irreducible representations (IRs) of C_{3v} and TR symmetries. A_1, A_2 and E are IRs of C_{3v} and $\pm$ are parity under TR. $\tau_\pm = (\tau_0 \pm \tau_z)/2$.

those symmetry operations, according to the convention in Supplementary Ref. [15], are represented as

$$
\begin{aligned}
C_3 &\doteq \begin{pmatrix} e^{-\frac{i\pi}{3}} & 0 & 0 & 0 \\ 0 & -1 & 0 & 0 \\ 0 & 0 & e^{\frac{i\pi}{3}} & 0 \\ 0 & 0 & 0 & -1 \end{pmatrix} \\
m_{1\bar{1}0} &\doteq -i\sigma_x\tau_0 = \begin{pmatrix} 0 & 0 & -i & 0 \\ 0 & 0 & 0 & -i \\ -i & 0 & 0 & 0 \\ 0 & -i & 0 & 0 \end{pmatrix} \\
\mathcal{T} &\doteq -i\sigma_y\tau_0 = \begin{pmatrix} 0 & 0 & -1 & 0 \\ 0 & 0 & 0 & -1 \\ 1 & 0 & 0 & 0 \\ 0 & 1 & 0 & 0 \end{pmatrix}\mathcal{K} ,
\end{aligned}
\tag{46}
$$

where τ's and σ's are Pauli matrices for E_1, H_1 indexes and $\pm$ indexes, respectively. According to the symmetry representations, the matrix and momenta of the effective model can be classified as Supplementary Tab. **5**.

From Supplementary Tab. **5**, the most general symmetry-allowed Hamiltonian without the electron-strain coupling can be derived to the k^2 order, resulting in Supplementary Eq. (40). As shown in Supplementary Tab. **5**, u_{ij} behaves the same as the k^2 term, and thereby the electron-strain coupling has the same form as the k^2 term in Supplementary Eq. (40).

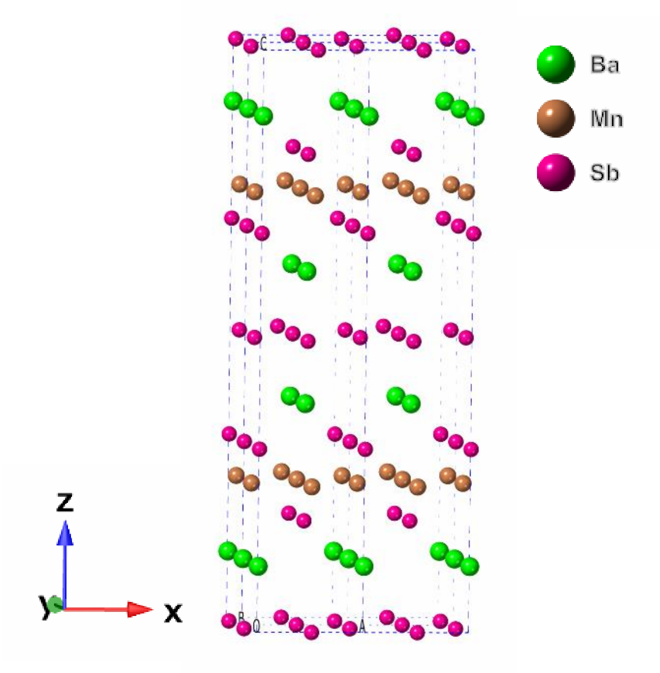

Supplementary Fig. 5. The crystalline structure of $BaMnSb_2$ generated from CrystalMaker.

Supplementary Note 6. $BaMnSb_2$

In this section, we include more details on $BaMnSb_2$.

A. Review

In this part, we review the form and the dispersion of the TB model derived in Supplementary Ref. [17] for integrity. This part does not contain any original results. More details can be found in Supplementary Ref. [17].

According to the main text, there are two Sb atoms in one unit cell, labeled as 1 and 2, that have sub-lattice vectors $\boldsymbol{\tau}_1 = (x_1 a, 0)$ and $\boldsymbol{\tau}_2 = (x_2 a, b/2)$, respectively. a, b are the lattice constants of the unit cell in x, y direction and the values of $x_{1,2}$ are given later. Combined with p_x and p_y orbitals, the bases of the TB model are $|\mathbf{R}+\boldsymbol{\tau}_i, \alpha, s\rangle$ with the lattice vector $\mathbf{R} = (l_x a, l_y b)$ $(l_{x,y} \in \mathbb{Z})$, the sublattice index $i = 1, 2$, the orbital index $\alpha = p_x, p_y$, and the spin-z index $s =\uparrow, \downarrow$. The TB model consists of the on-site term H_0, the nearest-neighboring (NN) hopping H_1 and the next-NN hopping H_2 in the TB model, *i.e.* $H_{TB} = H_0 + H_1 + H_2$. H_0 has the form

$$H_0 = \sum_{\mathbf{R},i} c^\dagger_{\mathbf{R}+\boldsymbol{\tau}_i} M_i c_{\mathbf{R}+\boldsymbol{\tau}_i} \tag{47}$$

with

$$c^\dagger_{\mathbf{R}+\boldsymbol{\tau}_i} = (c^\dagger_{\mathbf{R}+\boldsymbol{\tau}_i,p_x,\uparrow}, c^\dagger_{\mathbf{R}+\boldsymbol{\tau}_i,p_x,\downarrow}, c^\dagger_{\mathbf{R}+\boldsymbol{\tau}_i,p_y,\uparrow}, c^\dagger_{\mathbf{R}+\boldsymbol{\tau}_i,p_y,\downarrow}) \ . \tag{48}$$

H_1 reads

$$H_1 = \sum_{\mathbf{R}} \sum_{n=1}^{4} c^\dagger_{\mathbf{R}+\Delta\mathbf{R}_n+\boldsymbol{\tau}_2} T_n c_{\mathbf{R}+\boldsymbol{\tau}_1} + h.c. \ , \tag{49}$$

where $\Delta\mathbf{R}_1 = (0, 0)$, $\Delta\mathbf{R}_2 = (a, 0)$, $\Delta\mathbf{R}_3 = (a, -b)$ and $\Delta\mathbf{R}_4 = (0, -b)$. H_2 reads

$$H_2 = \sum_{\mathbf{R},i} \sum_{n=x,y} c^\dagger_{\mathbf{R}+\Delta\mathbf{R}_n+\boldsymbol{\tau}_i} Q_{ni} c_{\mathbf{R}+\boldsymbol{\tau}_i} + h.c. \ , \tag{50}$$

where $\Delta\mathbf{R}_x = (a, 0)$ and $\Delta\mathbf{R}_y = (0, b)$. The forms of M's, T's, and Q's are

$$\begin{aligned}
&M_1 = \widetilde{m}_0\tau_0\sigma_0 + \widetilde{m}_1\tau_z\sigma_0 + \lambda_0\tau_y\sigma_z \ , \\
&M_2 = C_{4z}^{OS} M_1 (C_{4z}^{OS})^\dagger \ , \\
&T_1 = t_0\tau_0\sigma_0 + t_1\tau_x\sigma_0 + it_2\tau_y\sigma \ , T_2 = \frac{\tau_z\sigma_y T_1 \tau_z\sigma_y}{f(\alpha)} \ , \\
&T_4 = \tau_z\sigma_y T_1 \tau_z \sigma_y \ , \ T_3 = \frac{T_1}{f(\alpha)} \ , \\
&Q_{x1} = t_3\tau_0\sigma_0 + t_4\tau_z\sigma_0 \ , \ Q_{x2} = t_5\tau_0\sigma_0 + t_6\tau_z\sigma_0 \ , \\
&Q_{y1} = C_{4z}^{OS} Q_{x2} (C_{4z}^{OS})^\dagger \ , \ Q_{y2} = C_{4z}^{OS} Q_{x1} (C_{4z}^{OS})^\dagger \ ,
\end{aligned} \tag{51}$$

where $f(\alpha) = 0.2\alpha + 1$, and $C_{4z}^{OS} = (-\mathrm{i}\tau_y) e^{-\mathrm{i}\frac{\sigma_z}{2}\frac{\pi}{2}}$ is the representation of the four-fold rotation along z in the orbital and spin subspace. α is the dimensionless distortion parameter; $\alpha = 0$ and $\alpha = 1$ correspond to the non-distorted and fully distorted cases, respectively. Moreover, the distortion effect on the relative atom positions is chosen as $x_1 = \frac{1}{2} + (0.4512 - \frac{1}{2})\alpha$ and $x_2 = 0.01729\alpha$, while we neglect distortion-induced change of a and b.

The numerical calculation is done for

$$\begin{aligned}
&\widetilde{m}_0 = 0 \ , \ \widetilde{m}_1 = 0.3\text{eV} \ , \ \lambda_0 = 0.25eV \ , \ t_0 = 1\text{eV} \ , \\
&t_1 = 2\text{eV} \ , \ t_2 = 0 \ , \ t_3 = 0.1\text{eV} \ , t_4 = -0.06\text{eV} \ , \\
&t_5 = 0.15\text{eV} \ , \ t_6 = -0.06\text{eV} \ , \ \text{and } a = b = 4.5\text{Å} \ .
\end{aligned} \tag{52}$$

The energy dispersion for $\alpha = 1$ is shown in the supplementary material of Supplementary Ref. [17].

B. TB Calculation of PET

The main effect of the strain in the TB model is to change the hopping amplitudes among atoms[18,19], which can be modeled by performing the following replacement[18] to the hopping parameters:

$$t_{ab} \to \left(1 - \beta \frac{\delta_i \delta_j u_{ij}}{|\boldsymbol{\delta}|^2}\right) t_{ab} \tag{53}$$

, where t_{ab} is the hopping parameter between atoms at $\mathbf{r}_a$ and $\mathbf{r}_b$ in the non-deformed case, and $\boldsymbol{\delta} = \mathbf{r}_a - \mathbf{r}_b$. β is the electron-phonon coupling parameter whose value for $BaMnSb_2$ has not been determined, and thereby we adopt the typical value $\beta = 2$ for the TMDs[18] to give a reasonable estimation of the PET jump.

C. Effective Model Analysis

To analytically demonstrate the PET jump, we project the tight-binding model into the subspace spanned by two degenerate states at each gap closing point (valley). As discussed in Supplementary Ref. [17], the resultant effective model reads

$$h_\pm^{(0)}(\mathbf{q}) = (E_0 \pm v_0 q_y)\tau_0 \pm v_2 q_y \tau_z \pm v_1 q_x \tau_x \pm (E_1 + \lambda)\tau_y \ , \tag{54}$$

where $h_\pm(\mathbf{q})$ is around $\mathbf{K}_\pm$ with $\mathbf{q} = \mathbf{k} - \mathbf{K}_\pm$, the two bases of $h_+^{(0)}$ ($h_-^{(0)}$) are $|\mathbf{K}_+, p_x \pm \mathrm{i}p_y, \uparrow\rangle$ ($|\mathbf{K}_-, p_x \pm \mathrm{i}p_y, \downarrow\rangle$), and the term with small coefficient has been omitted. E_1 and v_1 are given by the distortion, λ labels the SOC strength, and we choose $E_1 < 0, \lambda > 0$ without loss of generality. According to Supplementary Eq. (54), the gap closing can be achieved by tuning the distortion parameter E_1 to $E_1 + \lambda = 0$, which changes the Z_2 index since only one Dirac cone appears in half 1BZ. To study the PET jump, we include the electron-strain coupling with the form

$$h_\pm^{(1)}(\mathbf{q}) = N_0\tau_0 + N_1\tau_x + N_2\tau_z \ , \tag{55}$$

where $N_1 = \xi_{xy}(u_{xy} + u_{yx})$ and $N_i = \xi_{i,xx}u_{xx} + \xi_{i,yy}u_{yy}$ for $i = 0, 2$. It is derived from the symmetry consideration and the fact that the τ_y term is valley-dependent and thus of higher order. Combining the above equation with

Supplementary Eq. (54), we obtain the non-zero PET jump

$$\begin{aligned}\Delta\gamma_{xxx} &= -e\frac{\text{sgn}(v_1v_2)}{\pi v_2}\xi_{2,xx}\\ \Delta\gamma_{xyy} &= -e\frac{\text{sgn}(v_1v_2)}{\pi v_2}\xi_{2,yy}\\ \Delta\gamma_{yxy} &= \Delta\gamma_{yyx} = e\frac{\text{sgn}(v_1v_2)}{\pi v_1}\xi_{xy}\ ,\end{aligned} \tag{56}$$

as E_1 is tuned from $-\lambda+0^-$ to $-\lambda+0^+$. Therefore, the gap closing and the PET jump are consistent with result for scenario (iii) of $p1m1$.

* cxl56@psu.edu

[1] D. Vanderbilt, Journal of Physics and Chemistry of Solids **61**, 147 (2000).
[2] S. Park and B.-J. Yang, Phys. Rev. B **96**, 125127 (2017).
[3] S. Murakami, S. Iso, Y. Avishai, M. Onoda, and N. Nagaosa, Phys. Rev. B **76**, 205304 (2007).
[4] F. Zhang, A. H. MacDonald, and E. J. Mele, Proceedings of the National Academy of Sciences **110**, 10546 (2013).
[5] H. Rostami, F. Guinea, M. Polini, and R. Roldán, npj 2D Materials and Applications **2**, 15 (2018).
[6] C. Fang and L. Fu, Phys. Rev. B **91**, 161105 (2015).
[7] L. Fu and C. L. Kane, Phys. Rev. B **76**, 045302 (2007).
[8] J. Ahn and B.-J. Yang, Phys. Rev. Lett. **118**, 156401 (2017).
[9] J. Li, A. F. Morpurgo, M. Büttiker, and I. Martin, Phys. Rev. B **82**, 245404 (2010).
[10] B. A. Bernevig, T. L. Hughes, and S.-C. Zhang, Science **314**, 1757 (2006).
[11] M. König, S. Wiedmann, C. Brüne, A. Roth, H. Buhmann, L. W. Molenkamp, X.-L. Qi, and S.-C. Zhang, Science **318**, 766 (2007).
[12] J. Li and K. Chang, Applied Physics Letters **95**, 222110 (2009).
[13] D. G. Rothe, R. W. Reinthaler, C.-X. Liu, L. W. Molenkamp, S.-C. Zhang, and E. M. Hankiewicz, New Journal of Physics **12**, 065012 (2010).
[14] E. G. Novik, A. Pfeuffer-Jeschke, T. Jungwirth, V. Latussek, C. R. Becker, G. Landwehr, H. Buhmann, and L. W. Molenkamp, Phys. Rev. B **72**, 035321 (2005).
[15] R. Winkler, S. Papadakis, E. De Poortere, and M. Shayegan, *Spin-Orbit Coupling in Two-Dimensional Electron and Hole Systems*, Vol. 41 (Springer, 2003) pp. 211–223.
[16] J. P. Laurenti, J. Camassel, A. Bouhemadou, B. Toulouse, R. Legros, and A. Lusson, Journal of Applied Physics **67**, 6454 (1990).
[17] J. Liu, J. Yu, J. Ning, L. Miao, L. Min, K. Lopez, Y. Zhu, H. Yi, T. Pillsbury, Y. Zhang, *et al.*, arXiv preprint arXiv:1907.06318 (2019).
[18] L. Li, E. V. Castro, and P. D. Sacramento, Phys. Rev. B **94**, 195419 (2016).
[19] Y. Wang, Z. Wang, J. Li, J. Tan, B. Wang, and Y. Liu, Phys. Rev. B **98**, 125402 (2018).
